# Physical activity, health, and life satisfaction: Four panel studies demonstrate reciprocal effects

**DOI:** 10.1111/aphw.70027

**Published:** 2025-04-14

**Authors:** Daniel Groß, Carl‐Walter Kohlmann

**Affiliations:** ^1^ University of Education Schwäbisch Gmünd Schwäbisch Gmünd Germany; ^2^ Zentrum für Psychiatrie Reichenau Reichenau Germany

**Keywords:** exercise, health, life satisfaction, long‐standing health issues, physical activity, well‐being

## Abstract

We examined the between‐person correlations and within‐person reciprocal effects of physical activity, long‐standing health issues, self‐rated health, and life satisfaction across four panels using random intercept cross‐lagged panel models. Data were analyzed from the Household, Income and Labour Dynamics in Australia Survey (HILDA, *N* = 32,913, 21 waves, 1‐year intervals), the German Socio‐Economic Panel (SOEP, *N* = 83,844, 14 waves, 2‐year intervals), the Dutch Longitudinal Internet Studies for the Social Sciences Panel (LISS, *N* = 14,778, 11 waves, 1‐year intervals), and the United Kingdom Household Longitudinal Study (UKHLS, *N* = 50,032, 4 waves, 2‐year intervals). The analysis of the first two panels focused on moderate‐to‐vigorous physical activity and sports participation in leisure time, whereas the latter two examined physical activity intensities (light, moderate, and vigorous). Across all panels, physical activity and its intensities were positively correlated with long‐standing health issues, self‐rated health, and life satisfaction. Within‐person analyses revealed primarily positive bidirectional effects between physical activity and self‐rated health and between physical activity and life satisfaction, with self‐rated health effects more pronounced at higher physical activity intensities and life satisfaction at lower intensities. Effects between physical activity and long‐standing health issues were less consistent, appearing mainly for moderate‐to‐vigorous physical activity intensities in 1‐year intervals. Physical activity intensities had different effects on self‐rated health and life satisfaction, and the effects were bidirectional in nature. These results suggest that physical activity interventions should be tailored to intensity: Light activity may enhance more effective life satisfaction, while higher intensities better support health. The existing bidirectional effects may further trigger an upward spiral, reinforcing improvements in both health and well‐being.

## INTRODUCTION

Research emphasizes the significant role of physical activity in promoting longevity and overall health, with even light activity providing notable benefits (e.g. Banach et al., 2023; Lear et al., 2017; Reiner et al., 2013). For mental illness, moderate‐to‐vigorous physical activity is particularly effective, often yielding greater benefits than lighter activity (Singh et al., 2023). Interestingly, recent studies reveal a bidirectional relationship: Whereas physical activity promotes health, an increase in health—including brain health and a reduction in mental illness—can also predict increased physical activity, with an increase in mental health often associated with higher intensity levels (Casanova et al., 2023; Rodriguez‐Ayllon et al., 2024; Zhao et al., 2023). It should be noted that symptoms of mental illness and well‐being are empirically not on a single continuum but instead show negative and moderate correlations (Keyes, 2002). Physical activity has also shown a positive impact on well‐being, even at lower intensities (e.g. Buecker et al., 2021; Timm et al., 2024; Wiese et al., 2018). Well‐being is commonly understood through two main approaches (Ryan & Deci, 2001): *hedonic well‐being*, which involves life evaluation through affective (positive and negative) and cognitive (life satisfaction) components, often referred to as subjective well‐being (Diener, 1984); and *eudaimonic well‐being*, which focuses on meaning, self‐realization, and vitality. As part of eudaimonic well‐being, vitality reflects the “feeling of aliveness and energy” (Ryan & Frederick, 1997, p. 529) and highlights the importance of being fully engaged and functioning optimally (Ryan & Deci, 2001). Joshanloo (2018) found that eudaimonic well‐being was significantly correlated with life satisfaction (*r* = .276), positive affect (*r* = .373), and negative affect (*r* = −.163). However, more precise examinations of associations between physical activity and well‐being have revealed nuanced findings. Studies on affective components show that bidirectional associations between physical activity and well‐being vary widely, often analyzed in shorter intervals, as momentary feelings such as positive and negative affect fluctuate throughout the day (Timm et al., 2024). By contrast, life satisfaction tends to be more stable over time (Luhmann et al., 2021). Research consistently shows positive effects of physical activity on life satisfaction across varying intervals, including 1‐ and 2‐year spans (Buecker et al., 2021; Kuper et al., 2023; Schmiedeberg & Schröder, 2017; Wiese et al., 2018). Studies exploring whether life satisfaction, in turn, predicts physical activity are limited. A study by Kim et al. (2021) indicated that higher life satisfaction positively affected physical activity over a 4‐year period, though it did not examine the reciprocal effect within the same timeframe.

Regarding eudaimonic well‐being, specifically the relationship between physical activity and a sense of meaning, findings are mixed. Pfund et al. (2022) reported that only moderate (but not vigorous) physical activity was bidirectionally associated with meaning in life across five monthly intervals. By contrast, Yemiscigil and Vlaev (2021) found bidirectional associations with both moderate and vigorous physical activity over 4‐ and 9‐year intervals. These differences may reflect variations in study design: Pfund et al. (2022) focused on within‐person effects, whereas Yemiscigil and Vlaev (2021) analyzed between‐person effects. Focusing on vitality, a recent systematic literature review of everyday‐life studies by Timm et al. (2024) indicated a strong consistent physical activity link at the within‐person level with feelings of energy in both directions.

Focusing on self‐rated health—a multidimensional measure encompassing physical, mental, and social well‐being (Diener et al., 1999; Hamplová et al., 2022)—its relationship with physical activity is frequently examined using cross‐sectional designs, and relatively few studies have differentiated physical activity by intensity. Self‐rated health often aligns with objective health metrics, particularly in the general population (Wu et al., 2013). Yet, it does not always correspond to the absence of disease or physical limitations, especially in older adults (Wettstein et al., 2016). However, changes in functioning, illness, and pain are critical determinants of fluctuations in self‐rated health for both men and women (Lazarevič & Quesnel‐Vallée, 2023). As a global indicator of health status, self‐rated health captures a broad spectrum of factors, including disease prevalence, laboratory measures, and other health‐related variables (Wu et al., 2013).

Studies that account for the intensity of physical activity consistently find that higher intensity activity is associated with better self‐rated health (Galán et al., 2010; Rosenkranz et al., 2013; von Rosen & Hagströmer, 2019). Longitudinal research further supports this relationship, demonstrating that increases in physical activity correspond to improvements in self‐rated health over time, with effects documented across intervals ranging from 2 weeks to 10 years (Barone Gibbs et al., 2021; Cheval et al., 2021; Oftedal et al., 2021). By contrast, the reverse effect—self‐rated health predicting subsequent physical activity—has been explored less frequently and typically over longer periods. For instance, Barone Gibbs et al. (2021) found that lower self‐rated health predicted declines in moderate‐to‐vigorous physical activity across a 10‐year period, with no effect on light physical activity. Studies reporting counterevidence, such as a lack of association or the finding that higher self‐rated health was unexpectedly linked to decreased physical activity, are rare and generally limited to longer intervals (8 and 10 years; Wong & Gong, 2023).

Distinguishing within‐person from between‐person effects is crucial, as they can reveal contrasting insights. For example, individuals who are generally more physically active (between‐person level) often report higher life satisfaction and better self‐rated health overall (An et al., 2020; Rosenkranz et al., 2013). However, within‐person evidence may show that periods of intense activity can temporarily lower an individual's self‐rated health due to fatigue or even, over time, the risk of injuries. Similarly, life satisfaction might dip immediately after an individual increases physical activity if it disrupts their routine or adds stress to an already busy schedule. This distinction underscores that while regular physical activity is generally linked to long‐term improvements in life satisfaction and self‐rated health, within‐person fluctuations in these outcomes may arise from the short‐term demands or cumulative stresses associated with increased activity (Curran & Bauer, 2011).

## THE PRESENT STUDIES

In four panel studies, we aimed to simultaneously examine both general between‐person associations and within‐person cross‐lagged relationships between physical activity (at varying intensities: light, moderate, and vigorous) and outcomes related to long‐standing health issues, subjective health, and life satisfaction across 1‐ and 2‐year intervals.

We specifically focused on the cognitive aspect of hedonic well‐being—namely, life satisfaction—for two reasons. First, life satisfaction is relatively stable over time (Luhmann et al., 2021), whereas feelings of positive and negative affect are highly variable, often fluctuating within a day and are better analyzed in shorter intervals (Timm et al., 2024). Second, eudaimonic well‐being (e.g. meaning in life or vitality) could not be included, as it was inconsistently measured across the four panel studies.

We applied random intercept cross‐lagged panel models (RI‐CLPMs) to clarify the prospective within‐person effects, an approach that fills a gap in the existing literature, which has largely concentrated on isolated, unidirectional relationships, or focused primarily on between‐person associations over extended intervals. In RI‐CLPMs, lagged effects examine whether deviations from an individual's baseline in one construct (e.g. physical activity) predict deviations in another construct (e.g. self‐rated health) at subsequent time points. A positive lagged effect suggests that deviations in one variable (either up or down) are followed by similar deviations in another variable, while a negative effect suggests opposite changes (Mulder & Hamaker, 2021). Additionally, the random intercept factors in RI‐CLPMs reflect between‐person associations over multiple waves (Hamaker et al., 2015), allowing us to explore physical activity's relationships with health and life satisfaction at both within‐ and between‐person levels.

Between‐person correlations reveal whether higher intensities of physical activity are generally associated with better outcomes for long‐standing health issues, self‐rated health, and life satisfaction, which we anticipate observing. Conversely, within‐person effects indicate how individual changes in physical activity predict changes in these outcomes over time, and vice versa. We hypothesize that within‐person increases in physical activity will be positively associated with future increases in long‐standing health issues, self‐rated health, and life satisfaction. Likewise, improvements in these health outcomes should positively influence future physical activity. Regarding the intensity of physical activity, we expect, based on findings by Wolf and Wohlfart (2014) among Australian national park visitors, that lighter physical activity will be prospectively more strongly associated with life satisfaction, whereas more intense physical activity will be more closely associated with long‐standing health issues and self‐rated health. Wolf and Wohlfart (2014) found, albeit at the between‐person level, that walkers, hikers, and runners reported improvements in well‐being and self‐rated health, with walkers and hikers experiencing more pronounced gains in well‐being than in self‐rated health. Accordingly, we anticipate that at the within‐person level, lighter physical activity will show a stronger association with life satisfaction, whereas more intense activity will be more closely linked to health outcomes. Finally, the question of whether a within‐person increase in life satisfaction also predicts a subsequent increase in lighter physical activity (or whether improvements in long‐standing health issues or self‐rated health also prospectively lead to more intense physical activity) remains exploratory in nature.

## GENERAL METHOD

### Panel data, research model, and variables

**FIGURE 1:**
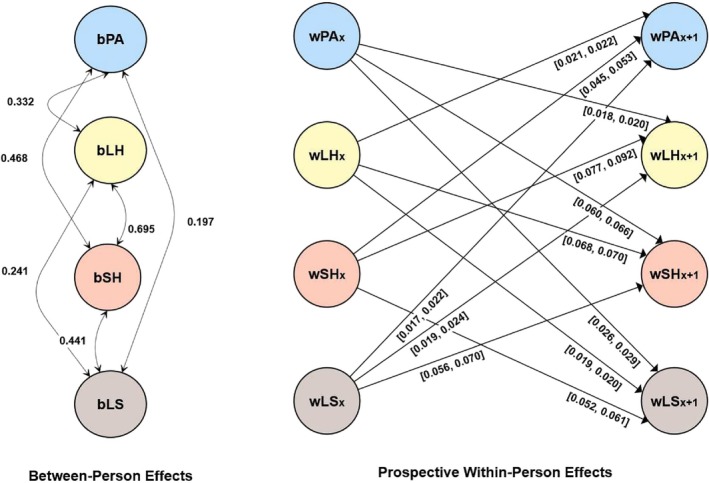
Results for the random intercept cross‐lagged panel model in Study 1 (Household, Income and Labour Dynamics in Australia [HILDA]). Only significant between‐person and cross‐lagged effects are shown for simplicity. The prefix b denotes between‐person effects. The prefix w denotes within‐person effects. [], describes the values between which the standardized effects vary; LH, long‐standing health condition; LS, life satisfaction. Standardized estimates are reported; PA, moderate‐to‐vigorous physical activity; SH, self‐rated health.

We tested the associations in four different panel studies with a total of 181,567 individuals: the Household, Income and Labour Dynamics in Australia (HILDA; Watson & Wooden, 2012; Study 1) Survey; the German Socio‐Economic Panel (SOEP; Goebel et al., 2019; Study 2), the Dutch Longitudinal Internet Studies for the Social Sciences (LISS; Scherpenzeel & Das, 2010; Study 3) Panel, and the United Kingdom Household Longitudinal Study (UKHLS; University of Essex, 2023; Study 4). With the first two panels, HILDA and SOEP, we tested the associations of moderate‐to‐vigorous physical activity and sports participation in leisure time^i^ with long‐standing health issues, self‐rated health, and life satisfaction, calculated as a “fourthvariate” RI‐CLPM. With the HILDA (22 waves from 2001 to 2022), we tested the associations in 1‐year intervals, and with the SOEP (14 waves from 1995 to 2021), we tested the associations in 2‐year intervals.^ii^ With the other two panels, LISS and UKHLS, we analyzed the associations of different physical activity intensities (light, moderate, and vigorous) in more detail, calculating “sixvariate” RI‐CLPMs. With the LISS (11 waves from 2008 to 2018), we tested the associations in 1‐year intervals, and with the UKHLS (four waves from 2015/16 to 2021/2022), we tested the associations in 2‐year intervals.^iii^ For an overview of the panel studies, see Table 1. Figure 1 in the ESM illustrates the general RI‐CLPM approach using only two variables (physical activity and long‐standing health issues) for the sake of visual simplicity.

Physical activity was measured in Study 1 with the following item: “In general, how often do you participate in moderate or intensive physical activity for at least 30 minutes?” In Study 2, sports participation in leisure time was measured with the following item: “Now some questions about your leisure time. Please indicate how often you participate in sports.” In Study 3, light physical activity was measured with the following item: “If you look back on the last 7 days, on how many of those days did you spend at least 10 minutes walking?” and in Study 4 with: “Now think about the time you spent walking in the last 7 days.” In Study 3, moderate physical activity was measured with the following item: “Think of activities that you performed over the last 7 days that require moderate physical exertion” and in Study 4 with: “Now think about activities that take moderate physical effort that you did in the last 7 days.” In Study 3, vigorous physical activity was measured with: “If you look back on the last 7 days, on how many of those days did you perform a strenuous physical activity such as lifting heavy loads, digging, aerobics, or cycling?” and in Study 4 with: “Now, think about all the vigorous activities that take hard physical effort that you did in the last 7 days.” In all four panels, the self‐rated health variable asked about the participant's general state of health, and the life satisfaction variable asked about their general satisfaction with life as a whole. The long‐standing health issue variable differed slightly between the panels, as there was no common variable available (i.e. a long‐standing health issue that lasted for 6 months or more [HILDA, Study 1] or that lasted at least 12 months [UKHLS, Study 4], sick leave from work for more than 6 weeks [SOEP, Study 2], or suffering from any kind of long‐standing disease [LISS, Study 3]; for detailed descriptions of these variables, see Section 3 of each study in the ESM). However, two of the four panels included “long‐standing” directly in the question (LISS, UKHLS), whereas one panel used “long‐term” (HILDA), and another did not reference either term (SOEP). Overall, the questions focused primarily on long‐standing illnesses that differed slightly in their time span and description. However, responses were recoded so that higher values indicate better long‐standing health issues. In general, there is no universal definition of long‐standing health issues, not even by the WHO, leading to variations in how it is understood by different organizations, countries, and researchers. Exact descriptions of all variables and detailed information on age and gender ratios at each time point for each panel study can be found in Table 2 in the ESM. Descriptives and distributions for physical activity, long‐standing health issues, self‐rated health, and life satisfaction at all time points and all panels can be found in Tables 3–6 in the ESM.

### Data analysis and interpretation

All analyses were performed in R (Version 4.4.0). For the RI‐CLPM calculation, we used robust maximum likelihood estimation (MLR) to account for the noncontinuous structure of the data. Missing data handling and the testing of the tenability of full stationarity constraints (i.e. time‐invariant autoregressive and cross‐lagged parameters and time‐invariant residual variances and covariances) are described in more detail in the ESM. We assumed full stationarity constraints for Studies 1, 3, and 4 and partial stationarity constraints (i.e. time‐invariant autoregressive and cross‐lagged parameters) for Study 2. The model fits were evaluated using the traditional fixed cutoffs of RMSEA < .06, SRMR ≤ .08, and CFI ≥ .95 (e.g. Schreiber et al., 2006). Cross‐lagged effect sizes were computed using standard estimates, with Orth et al. (2024) defining effect sizes of .03 as small, .07 as moderate, and .12 as large. However, it should be noted that these effect sizes are based on frequencies and their percentiles in the literature. For the random intercept factor correlations, which are similar to cross‐sectional correlations but are based on information from multiple waves, effect sizes of .10, .30, and .50 were defined as small, moderate, and large, respectively (Cohen, 1992). We report standardized effects in the figures. Standardized effects can still vary over time. This variability can still occur because standardization depends on the variances of the within‐component predictor and outcome, which are generally not constrained to remain constant over time, even if residual variances and covariances are constrained (Mulder, 2023). Therefore, the standardized lagged effects can vary even while the unstandardized lagged effects are constrained to be equal over time. We did not preregister the current analyses. R‐code for all panels is available at https://osf.io/h3zpw. The code for all RI‐CLPMs was adapted from Usami et al. (2019).

## STUDY 1: HILDA

### Results

A detailed description of the results, including the RI‐CLPM model parameter estimates, which all showed good fit, is presented in the ESM and in Table 7 in the ESM. Additionally, all significant between‐person and cross‐lagged effects are visually represented in Figure 1. The ESM contains tables with correlations for each variable over time: physical activity (Table 8), long‐standing health issues (Table 9), self‐rated health (Table 10), and life satisfaction (Table 11).

### Summary

As expected, the results showed that physical activity had positive within‐person reciprocal prospective associations with long‐standing health issues, self‐rated health, and life satisfaction. It should be noted that physical activity generally had larger associations with self‐rated health than with long‐standing health issues or life satisfaction. The same picture emerged at the correlational between‐person level (random intercept correlations). Additionally, bidirectional associations were found between long‐standing health issues, self‐rated health, and life satisfaction.

## STUDY 2: SOEP

### Results

**FIGURE 2:**
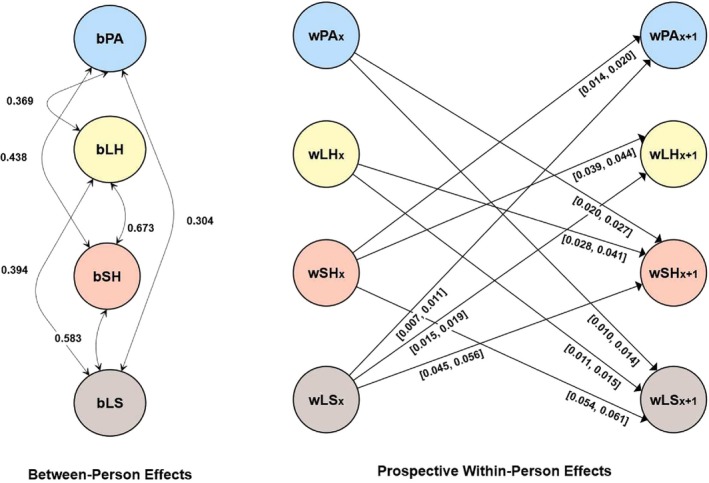
Results for the random intercept cross‐lagged panel model in Study 2 (Socio‐Economic Panel [SOEP]). Only significant between‐person and cross‐lagged effects are shown for simplicity. The prefix b denotes between‐person effects. The prefix w denotes within‐person effects. [], describes the values between which the standardized effects vary; LH, long‐standing health condition; LS, life satisfaction. Standardized estimates are reported; PA, sports participation in leisure time; SH, self‐rated health.

A detailed description of the results, including the RI‐CLPM model parameter estimates, which all showed good fit, is presented in the ESM and in Table 12 in the ESM. Additionally, all significant between‐person and cross‐lagged effects are visually represented in Figure 2. The ESM contains tables with correlations for each variable over time: physical activity (Table 13), long‐standing health issues (Table 14), self‐rated health (Table 15), and life satisfaction (Table 16).

### Summary

As expected, physical activity^iv^ had positive within‐person reciprocal prospective associations with self‐rated health and life satisfaction. These associations could also be seen at the correlational between‐person level. Here, a positive between‐person correlation between the random intercept of physical activity and the random intercept of long‐standing health issues also emerged. However, at the prospective within‐person level, there was no effect in either direction between physical activity and long‐standing health issues. Therefore, this finding is not in line with the anticipated prospective bidirectional relationship between physical activity and long‐standing health issues across this 2‐year period. Although the bidirectional associations between physical activity and self‐rated health and between physical activity and life satisfaction were both small, the bidirectional associations between physical activity and self‐rated health were twice as large as those between physical activity and life satisfaction. Additionally, bidirectional associations were found between long‐standing health issues, self‐rated health, and life satisfaction.

## STUDY 3: LISS

### Results

**FIGURE 3:**
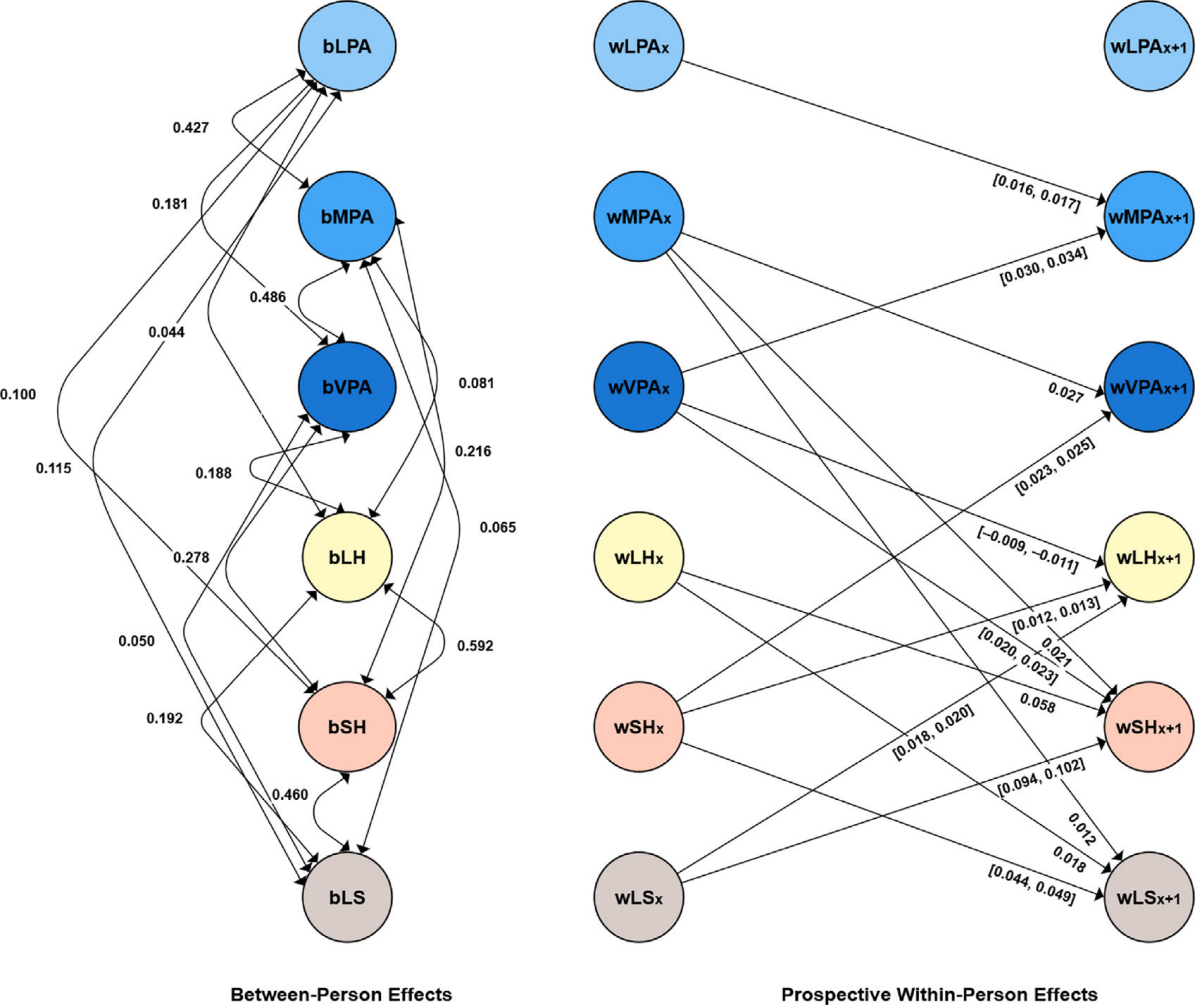
Results for the random intercept cross‐lagged panel model in Study 3 (Longitudinal Internet Studies for the Social Sciences [LISS]). Only significant between‐person and cross‐lagged effects are shown for simplicity. The prefix b denotes between‐person effects. The prefix w denotes within‐person effects. [], describes the values between which the standardized effects vary; LH, long‐standing health condition; LPA, light physical activity; LS, life satisfaction. Standardized estimates are reported; MPA, moderate physical activity; SH, self‐rated health; VPA, vigorous physical activity.

A detailed description of the results, including the RI‐CLPM model parameter estimates, which all showed good fit, is presented in the ESM and in Table 17 in the ESM. Additionally, all significant between‐person and cross‐lagged effects are visually represented in Figure 3. The ESM contains tables with correlations for each variable over time: light physical activity (Table 18), moderate physical activity (Table 19), vigorous physical activity (Table 20), long‐standing health issues (Table 21), self‐rated health (Table 22), and life satisfaction (Table 23).

### Summary

As expected, we found positive within‐person reciprocal prospective associations between vigorous physical activity and self‐rated health. Furthermore, there was an effect of moderate physical activity on self‐rated health. This finding means that the bidirectional effect between physical activity and self‐rated health was mainly evident at a higher intensity. This result supported not only the expectation that an increase in more intensive physical activity will be associated with a prospective increase in self‐rated health but also the reverse effect: that an increase in self‐rated health will be associated with a prospective increase in more intensive physical activity. Additionally, we found a positive effect of moderate physical activity on life satisfaction. This finding did not explicitly indicate that lighter physical activity has a positive effect on life satisfaction. We aimed to clarify whether light or vigorous physical activity has a more positive effect in more detail in Study 4. An inverse effect of life satisfaction on physical activity of any intensity was also not found in this study. For long‐standing health issues and physical activity, the results were not in line with our expectations. The results indicated only a negative effect of vigorous physical activity on long‐standing health issues. In this case, a negative effect means that an increase in individual vigorous physical activity was associated with a reduction in long‐standing health issues. There was also a positive bidirectional effect between moderate and vigorous physical activity, a finding that shows that both intensities can prospectively increase in frequency. Light physical activity also had a positive effect on moderate physical activity, although there was no reverse effect here. There was also a positive bidirectional effect between moderate and vigorous physical activity. Additionally, bidirectional associations were found between long‐standing health issues, self‐rated health, and life satisfaction. At the correlational, between‐person, random intercept level, all variables were positively correlated with each other, but this finding did not always hold at the prospective within‐person level, as just mentioned.

## STUDY 4: UKHLS

### Results

**FIGURE 4:**
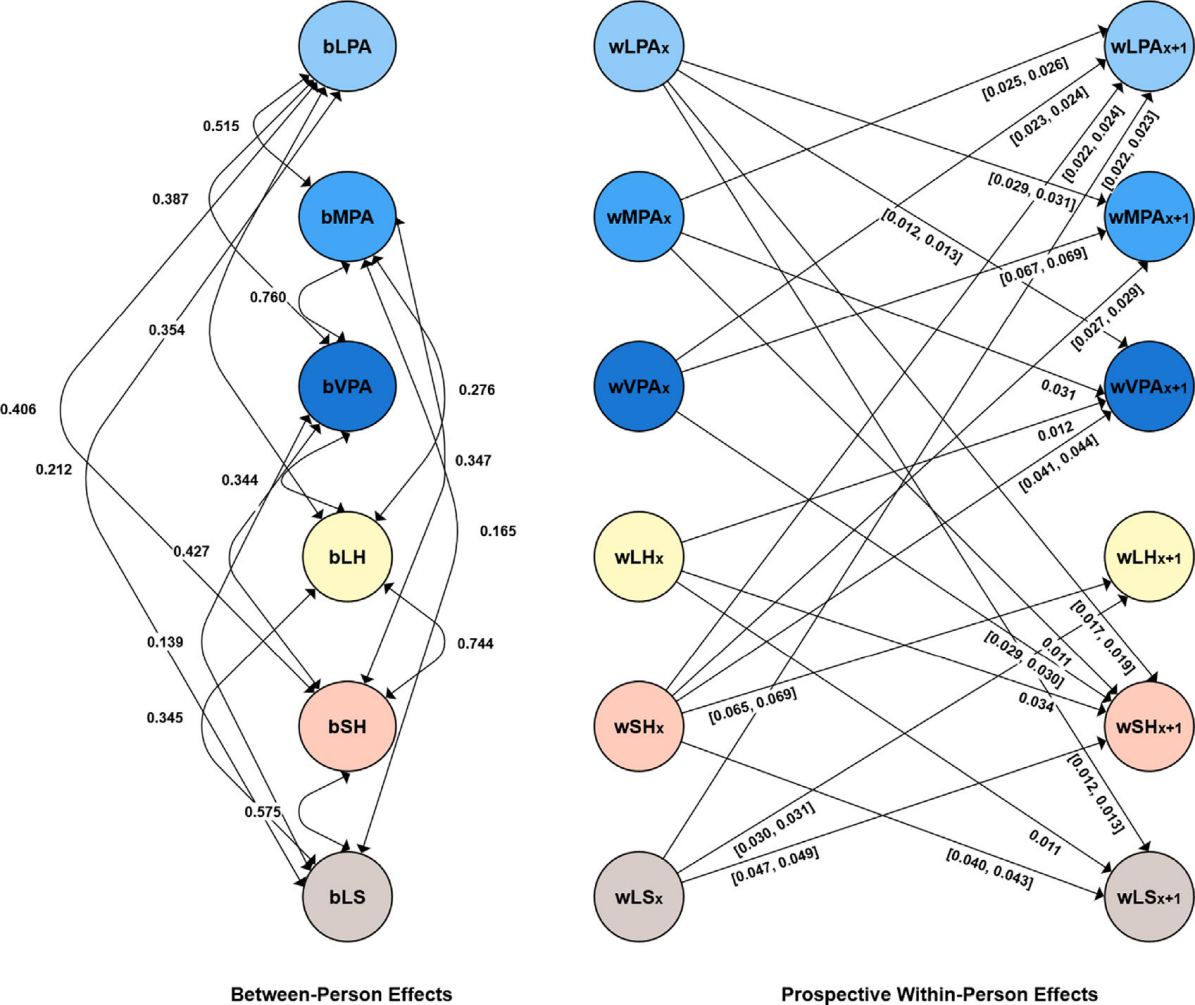
Results for the random intercept cross‐lagged panel model in Study 4 (UK (Understanding Society) Household Longitudinal Study [UKHLS]). Only significant between‐person and cross‐lagged effects are shown for simplicity. The prefix b denotes between‐person effects. The prefix w denotes within‐person effects. [], describes the values between which the standardized effects vary; LH, long‐standing health condition; LPA, light physical activity; LS, life satisfaction. Standardized estimates are reported; MPA, moderate physical activity; SH, self‐rated health; VPA, vigorous physical activity.

A detailed description of the results, including the RI‐CLPM model parameter estimates, which all showed good fit, is presented in the ESM and in Table 24 in the ESM. Additionally, all significant between‐person and cross‐lagged effects are visually represented in Figure 4. The ESM contains tables with correlations for each variable over time: light physical activity (Table 25), moderate physical activity (Table 26), vigorous physical activity (Table 27), long‐standing health issues (Table 28), self‐rated health (Table 29), and life satisfaction (Table 30).

### Summary

We found positive reciprocal within‐person prospective associations of all three intensities (light, moderate, and vigorous) with self‐rated health, with the effect size showing that the bidirectional associations were strongest for vigorous physical activity even though the associations were generally small. By contrast, the bidirectional relationship between physical activity and life satisfaction was more likely to be found at low intensity, as indicated by the bidirectional effect between light physical activity and life satisfaction. Therefore, these results were in accordance not only with the expectations that an increase in more intensive physical activity will be associated with an increase in self‐rated health and an increase in lighter physical activity will be associated with an increase in life satisfaction but also with the expectations that the reverse effects exist. When looking at long‐standing health issues and physical activity, only a positive effect of long‐standing health issues on vigorous physical activity emerged. This finding means that a deterioration in long‐standing health issues was associated with a prospective decline in vigorous physical activity. In addition, positive bidirectional associations were found between light, moderate, and intense physical activity, showing that all intensities had positive effects on each other. Additionally, bidirectional associations were found between long‐standing health issues, self‐rated health, and life satisfaction. At the correlational, between‐person, and random intercept level, all variables were positively correlated with each other, but this finding did not always hold at the prospective within‐person level, as just mentioned.

## GENERAL DISCUSSION

Past studies investigating the relationships between physical activity, health, and life satisfaction have often taken a narrow perspective, focusing mainly on isolated, unidirectional relationships between these variables at the between‐person level or considered longtime intervals of many years. However, there is a recognized need for more comprehensive research that delves into the nuanced interplay between these variables over time, with shorter intervals and within individuals. To address this gap, we utilized data from four large panel studies and analyzed them with RI‐CLPMs.

### Associations of physical activity with long‐standing health issues, self‐rated health, and life satisfaction

#### Between‐person associations

All panels indicated that physical activity as well as each intensity level was positively correlated with long‐standing health issues, self‐rated health, and life satisfaction. These findings mean that, in line with the literature (e.g. An et al., 2020; Rosenkranz et al., 2013), people who generally exercise more than others (during their leisure time; see Study 2), regardless of the intensity, have better health and are generally more satisfied with their lives. Focusing more precisely on these associations, Studies 1 and 2 indicated that the strength of associations increased from life satisfaction to long‐standing health issues to self‐rated health, and the effects were in the small to medium range. Looking at the different intensities in Studies 3 and 4, the correlation between light physical activity and life satisfaction was higher than between higher intensities of physical activity and life satisfaction. However, the overall effects were small. Study 3 (but not Study 4) also showed that physical activity's correlations with long‐standing health issues and self‐rated health increased as the levels of intensity of physical activity increased, although the overall effects were small, a trend that has also been found in other studies (e.g. Galán et al., 2010; von Rosen & Hagströmer, 2019).

#### Prospective within‐person associations

All panels revealed consistent positive bidirectional patterns between physical activity and self‐rated health, as well as between physical activity and life satisfaction, challenging the notion of unidirectional associations (e.g. Buecker et al., 2021; Cheval et al., 2021; Oftedal et al., 2021; Wiese et al., 2018). In essence, the findings underscore the nuanced and mutually reinforcing nature of the connections that physical activity has with self‐rated health and life satisfaction over time in a person. The examination of activity intensities unveiled positive bidirectional associations for lower activity levels and life satisfaction, primarily in Study 4. Thus, this result also fits with the finding of Pfund et al. (2022), who found a bidirectional relationship between physical activity and meaning in life for lower intensities of physical activity. Conversely, positive bidirectional relationships were found between physical activity and self‐rated health, especially at higher intensities, which is consistent with bidirectional findings between physical activity and mental/physical health (Barone Gibbs et al., 2021; Zhao et al., 2023). To sum it up, these results not only support the expectation that an increase in more intensive physical activity will be associated with an improvement in self‐rated health and that an increase in lighter physical activity will enhance life satisfaction but also demonstrate that reverse effects exist: specifically, that an increase in self‐rated health will primarily associated with an increase in higher physical activity intensities, and that an increase in life satisfaction will mainly associated with an increase in lighter physical activity. This observation also provided a potential explanation for the notable discrepancy in the size of bidirectional effects of physical activity with self‐rated health and life satisfaction in Studies 1 and 2. Whereas small positive bidirectional associations were evident between physical activity (moderate‐to‐vigorous activity and sports participation in leisure time) and self‐rated health, as well as between physical activity and life satisfaction, the bidirectional associations were approximately twice as large between physical activity and self‐rated health. The bidirectional effects of light physical activity on life satisfaction may stem from psychological processes, such as enhanced self‐efficacy, motivation, goal setting, and self‐regulation, which are known to influence engagement in health behaviors (Kim et al., 2021). Light physical activity, as it is easier to initiate and less demanding in terms of self‐regulation and stress, may also explain these findings. Supporting this idea, Soya et al. (2007) demonstrated in animal models that minimally stressful light physical activity may yield greater hippocampal benefits than more strenuous activity. As lower hippocampal volume has been linked to reduced well‐being (Van't Ent et al., 2017), this connection may be relevant, although a recent systematic review reported inconsistent neural correlates of well‐being (de Vries et al., 2023). Additionally, Otsuka et al. (2016) found that low‐speed running, but not high‐speed running, efficiently induces optimal neuronal activation with antidepressant and anxiolytic properties. Future research should further explore these mechanisms and refine our understanding of the bidirectional relationships between physical activity, self‐rated health, and life satisfaction. The prospective effects of physical activity on long‐standing health issues and vice versa did not line up with our expectations, as no consistent pattern emerged across the panels. It should be noted, however, that the assessment of long‐standing health issues varied slightly between the panels, and there is no universal definition of long‐standing health issues. Overall, the concept of long‐standing health issues remains fluid, shaped by varying criteria and contextual factors, although all panels focused on long‐standing health issues. Study 1 revealed a positive bidirectional within‐person association between moderate‐to‐vigorous physical activity and long‐standing health issues (that lasted for 6 months or more) at 1‐year intervals. However, Study 2, which focused on 2‐year intervals, did not identify a similar association for participation in sports during leisure time and long‐standing health issues (sick leave from work for more than 6 weeks). Besides the different definition of long‐standing health issues, this discrepancy can be partly attributed to the decreasing strength of the associations over longer observation periods, which was also found for self‐rated health and life satisfaction in Study 2 compared with Study 1 (Strain et al., 2020) or also to the fact that, in Study 2, physical activity was represented by participation in sports during leisure time, which does not reflect the totality of physical activity and is subject to a strongly subjective assessment of which activities can be identified as sports (Caspersen et al., 1985). Focusing on the different physical activity intensities in Studies 3 and 4 and long‐standing health issues, only two associations were found. With its 2‐year intervals, Study 4 indicated that a reduction in long‐standing health issues (that lasted at least 12 months) was followed by a reduction in vigorous physical activity, and Study 3 indicated an association that was not expected: A within‐person increase in vigorous physical activity had a negative prospective association with long‐standing health issues (“Do you suffer from any kind of long‐standing disease, affliction, or handicap, or do you suffer from the consequences of an accident?” [reverse‐scored]) 1 year later. Although the benefits of regular physical activity are well‐established, participation in primarily vigorous physical activity, which also includes competitive sports, is also associated with an increased risk of musculoskeletal injury and attrition (Garber et al., 2011). However, as the result here was rather unexpected, further studies should be conducted. Whereas the between‐person associations between physical activity and long‐standing health issues line up with findings on physical activity's associations with self‐rated health and life satisfaction, the prospective within‐person level results are less straightforward. Overall, with respect to prospective within‐person associations, rather small effects were observed. However, in principle, the associations between physical activity and health variables tend to be smaller when associations in both directions are considered (Strain et al., 2020). Furthermore, small effects are also important effects because they can accumulate into large effects over time (Funder & Ozer, 2019).

### Associations between long‐standing health issues, self‐rated health, and life satisfaction

In all four panels, a fairly consistent picture emerged between long‐standing health issues, self‐rated health, and life satisfaction. Initially, positive between‐person associations emerged between all three variables in all panels. Moreover, robust positive prospective within‐person bidirectional associations were evident between all three variables in all panels. These trends suggest that the results not only establish correlations between long‐standing health issues, self‐rated health, and life satisfaction at the between‐person level but also unveil a dynamic interplay wherein they mutually influence each other in either an upward or downward within‐person trajectory. Thus, the direction of association cannot be solely attributed to a unidirectional flow from one variable to another, as commonly discussed and found in prior studies (e.g. Diener & Chan, 2011; Gana et al., 2013; Kim et al., 2021).

### Associations between physical activity intensities (only Studies 3 and 4)

At the between‐person level, all three intensity levels were positively correlated with each other in both studies, with the highest correlations between moderate and vigorous physical activity and the lowest between light and vigorous physical activity. In both studies, positive bidirectional within‐person associations were also found between moderate and vigorous physical activity. This finding means that moderate physical activity is associated with a future increase in vigorous physical activity and vice versa, thus demonstrating that different intensity levels likely have positive influences on each other. Light physical activity also had a consistent positive effect on moderate physical activity. In sum, the results also suggest that starting with light physical activity increases the likelihood of engaging in more intense physical activity in the future.

### Practical and theoretical implications

These studies have implications for public health policies and further intervention studies, as they offer some advice regarding the intensity that should be chosen to achieve high levels of self‐rated health and life satisfaction. When the aim is to improve life satisfaction in the population, the recommendation is for people to be physically active at lower intensities, whereas higher intensity physical activities are the choice for improving health. Feedback effects of life satisfaction and health on the respective physical activity intensity can then set an upward spiral in motion. As just mentioned, one can also begin with light physical activity to increase health, as this starting point potentially promotes more intensive physical activity in the future, and life satisfaction also has a positive bidirectional effect with health.

### Strengths, limitations, and future directions

A significant strength of this study lies in its reliance on data from four large panels, spanning 1‐year intervals (Studies 1 and 3) and 2‐year intervals (Studies 2 and 4), while also considering various activity intensities. However, this design means that our conclusions are specific to these timeframes, precluding extrapolation to shorter or longer intervals. Even if a general picture emerges across the panels, it is important to realize that the lagged regression coefficients and the between‐person components critically depend on the time interval between the repeated measures (Kuiper & Ryan, 2018; Robitzsch & Lüdtke, 2024). Future studies could therefore systematically vary between longer and shorter intervals in order to get a clearer picture of the time interval at which the effects begin and the time interval at which they may weaken again. Moreover, between‐person variance may also arise from time‐varying covariates lacking stable components (Robitzsch & Lüdtke, 2024). Additionally, whereas our analysis provided valuable insights into the associations between physical activity, health, and life satisfaction, it did not delve deeply into the specific underlying processes driving these observed results. Moreover, the current analysis, which employed RI‐CLPMs, accounted exclusively for linear relationships, potentially overlooking nonlinear associations. Additionally, it should be noted that long‐standing health issues were recorded in binary form across all panels. Additionally, other variables were also not continuous, which means that treating ordinal data as continuous may introduce bias, even when robust MLR is used. However, previous research has suggested that it might be appropriate to treat ordinal data as continuous (e.g. Newsom & Smith, 2020; Robitzsch, 2020). Whereas an RI‐CLPM controls for time‐invariant covariates (e.g. gender), we did not account for the effect of time‐varying covariates, thus limiting causal interpretations. Moreover, the measurement questions and response formats of the variables in the panels sometimes differed. But the fundamental findings remained consistent. The variance of long‐standing health issues was limited, as we used only a yes (0) and no (1) answer format. Furthermore, this paper utilized single‐item self‐report measurements for all variables. Although such measures can provide valuable insights, they might not fully capture the multifaceted nature of the constructs. Using multiple‐item measurements for each construct could potentially yield more comprehensive and nuanced results, even though Cheung and Lucas (2014) showed that little validity was lost by using a single‐item measure compared with a multiple‐item measure of life satisfaction. While life satisfaction is generally stable over time (Luhmann et al., 2021), Bu et al. (2025) found that it fluctuates based on the time of day, day of the week, and season. Life satisfaction tends to peak in the morning and decline at night, with more pronounced variations on weekends than weekdays. Weekend peaks likely reflect the anticipation of leisure activities, whereas weekday fluctuations are smaller. Seasonal patterns also emerge, with life satisfaction being higher in all seasons compared to winter. These findings suggest that the timing of life satisfaction measurement—whether by time of day, weekday versus weekend, or season—may influence observed results. In addition, the nature of the physical activity variables in the HILDA and SOEP datasets is not entirely clear. Unlike the LISS and UKHLS panels, where questions about different activity intensities (light, moderate, and vigorous) were accompanied by examples, the HILDA survey began defining “moderate‐to‐vigorous physical activity” only in 2020. Before this year, it is unclear how respondents interpreted moderate‐to‐vigorous physical activity. However, the 2020 definition, which states that “moderate level physical activity will cause a slight increase in breathing and heart rate, such as brisk walking” does also not clearly differentiate between light and moderate‐to‐vigorous physical activity. Furthermore, in the SOEP, the definition of “sports participation in leisure time” is vague, relying on subjective interpretation. Consequently, future studies should explore whether objectively measured physical activity using accelerometers yields results similar to those obtained from self‐reported data. Last but not least, we did not analyze mediation effects in this study, for example, from physical activity to life satisfaction via self‐rated health. One reason is that there are multiple options for defining a direct or mediation effect, and it is not clear which option is appropriate. Future longitudinal studies could investigate such mediation effects in more detail, also because there are already indications that such effects exist (e.g. Downward et al., 2018). To this end, Downward et al. (2018) used structural equation models and also examined the effect in the other direction in separate models rather than in a joint model. Social capital was always taken into account as a further mediator.

### Conclusion

This comprehensive analysis examined the overall correlational between‐person and reciprocal within‐person dynamics of physical activity with long‐standing health issues, self‐rated health, and life satisfaction using data from four longitudinal panel studies. Across all panels, physical activity (including all intensities: light, moderate, and vigorous) was positively correlated with long‐standing health issues, self‐rated health, and life satisfaction at the within‐person bidirectional level, but bidirectional associations were primarily observed between physical activity and self‐rated health as well as between physical activity and life satisfaction. Prospective within‐person associations between physical activity and long‐standing health issues were less consistent and found primarily for moderate‐to‐vigorous physical activity in 1‐year intervals. Bidirectional associations between physical activity and self‐rated health were more pronounced at higher intensities, whereas bidirectional associations between physical activity and life satisfaction were more pronounced at lower intensities. These findings suggest that interventions should be tailored accordingly: light physical activity may be more effective in enhancing life satisfaction, while moderate‐to‐vigorous activity may yield greater health benefits. Leveraging these bidirectional effects could initiate an upward spiral, where improvements in health and life satisfaction reinforce further engagement in physical activity. This insight provides a valuable foundation for designing more targeted and effective interventions.

## CONFLICT OF INTEREST STATEMENT

Both authors declare no competing interests.

## ETHICS STATEMENT

For each of the four studies, informed consent was obtained from participants by the respective responsible institution. Ethical approval was not required for our research, as we analyzed existing and fully anonymized panel data.

## Supporting information


**Table S1.** Characteristics of the Four Panel Studies
**Table S2.** Age and Gender Ratios at Each Time Point
**Table S3.** Descriptives and Distributions for Physical Activity, Long‐Standing Health Issues, Subjective Health, and Life Satisfaction at All Time Points – HILDA Data
**Table S4.** Descriptives and Distributions for Physical Activity, Long‐Standing Health Issues, Subjective Health, and Life Satisfaction at All Time Points – SOEP Data
**Table S5.** Descriptives and Distributions for Light Physical Activity, Moderate Physical Activity, Vigorous Physical Activity, Long‐Standing Health Issues, Subjective Health, and Life Satisfaction at All Time Points – LISS Data
**Table S6.** Descriptives and Distributions for Light Physical Activity, Moderate Physical Activity, Vigorous Physical Activity, Long‐Standing Health Issues, Subjective Health, and Life Satisfaction at All Time Points – UKHLS Data
**Table S7.** Parameters and Model Fit Indices for the Random Intercept Cross‐Lagged Panel Model – HILDA Data
**Table S8.** Correlational Table of Moderate‐to‐Vigorous Physical Activity between the Different Measurement Points
**Table S9.** Correlational Table of Long‐Standing Health Issues between the Different Measurement Points
**Table S10.** Correlational Table of Self‐Rated Health between the Different Measurement Points
**Table S11.** Correlational Table of Life Satisfaction between the Different Measurement Points
**Table S12.** Parameters and Model Fit Indices for the Random Intercept Cross‐Lagged Panel Model – SOEP Data
**Table S13.** Correlational Table of Sport Participation between the Different Measurement Points
**Table S14.** Correlational Table of Long‐Standing Health Issues between the Different Measurement Points
**Table S15.** Correlational Table of Self‐Rated Health between the Different Measurement Points
**Table S16.** Correlational Table of Life Satisfaction between the Different Measurement Points
**Table S17.** Parameters and Model Fit Indices for the Random Intercept Cross‐Lagged Panel Model – LISS Data
**Table S18.** Correlational Table of Light Physical Activity between the Different Measurement Points
**Table S19.** Correlational Table of Moderate Physical Activity between the Different Measurement Points
**Table S20.** Correlational Table of Vigorous Physical Activity between the Different Measurement Points
**Table S21.** Correlational Table of Long‐Standing Health Issues between the Different Measurement Points
**Table S22.** Correlational Table of Self‐Rated Health between the Different Measurement Points
**Table S23.** Correlational Table of Life Satisfaction between the Different Measurement Points
**Table S24.** Parameters and Model Fit Indices for the Random Intercept Cross‐Lagged Panel Model – UKHLS Data
**Table S25.** Correlational Table of Light Physical Activity between the Different Measurement Points
**Table S26.** Correlational Table of Moderate Physical Activity between the Different Measurement Points
**Table S27.** Correlational Table of Vigorous Physical Activity between the Different Measurement Points
**Table S28.** Correlational Table of Long‐Standing Health Issues between the Different Measurement Points
**Table S29.** Correlational Table of Self‐Rated Health between the Different Measurement Points
**Table S30.** Correlational Table of Life Satisfaction between the Different Measurement Points
**Figure S1.** Statistical Model

## Data Availability

All analysis code is provided on the Open Science Framework (OSF) and can be retrieved from https://osf.io/h3zpw. On the OSF, we share all the code necessary to reproduce our results directly from the original datasets without the need for any additional steps to prepare the data. The datasets used in this study are made available through the respective data‐holding institutions. We are not allowed to make the data publicly available. This paper used unit record data from the Household, Income and Labour Dynamics in Australia Survey (HILDA) conducted by the Australian Government Department of Social Services (DSS), but the findings and views reported in this paper are those of the author[s] and should not be attributed to the Australian Government, DSS, or any of DSS' contractors or partners (10.26193/R4IN30). Additionally, this paper used data from the Socio‐Economic Panel (SOEP) made available by the German Institute for Economic Research (DIW), data from the Longitudinal Internet Studies for the Social Sciences (LISS) Panel administered by CentERdata (Tilburg University, the Netherlands), and data from the UK (Understanding Society) Household Longitudinal Study (University of Essex, Institute for Social and Economic Research).

## ENDNOTES

i It should be noted that physical activity is more than only sports in leisure time since it additionally includes occupational activities, conditioning exercises, household tasks (e.g. yardwork, cleaning, and home repair), and other activities (Caspersen et al., 1985).

ii In the SOEP, the four variables were only measured together regularly at 2‐year intervals from 1995 onwards, even if some of the variables were measured more frequently.

iii In the UKHLS, the six variables were measured together in yearly Waves 7, 9, 11, 12, and 13, beginning in 2015/2016. We used the data from Waves 7, 9, 11, and 13, to test the associations in 2‐year intervals.

iv Physical activity was measured here as participation in sports during leisure time. For more information, see the ESM.
