# Supplementary material for: Physical activity, health, and life satisfaction: Four panel studies demonstrate reciprocal effects
Source: Appl Psychol Health Well Being. 2025 Apr 14;17(2):e70027. doi: 10.1111/aphw.70027 (PMC11995367; doi:10.1111/aphw.70027)
Supplement: Supplementary file 1 — Table S1. Characteristics of the Four Panel Studies Table S2. Age and Gender Ratios at Each Time Point Table S3. Descriptives and Distributions for Physical Activity, Long‐Standing Health Issues, Subjective Health, and Life Satisfaction at All Time Points – HILDA Data Table S4. Descriptives and Distributions for Physical Activity, Long‐Standing Health Issues, Subjective Health, and Life Satisfaction at All Time Points – SOEP Data Table S5. Descriptives and Distributions for Light Physical Activity, Moderate Physical Activity, Vigorous Physical Activity, Long‐Standing Health Issues, Subjective Health, and Life Satisfaction at All Time Points – LISS Data Table S6. Descriptives and Distributions for Light Physical Activity, Moderate Physical Activity, Vigorous Physical Activity, Long‐Standing Health Issues, Subjective Health, and Life Satisfaction at All Time Points – UKHLS Data Table S7. Parameters and Model Fit Indices for the Random Intercept Cross‐Lagged Panel Model – HILDA Data Table S8. Correlational Table of Moderate‐to‐Vigorous Physical Activity between the Different Measurement Points Table S9. Correlational Table of Long‐Standing Health Issues between the Different Measurement Points Table S10. Correlational Table of Self‐Rated Health between the Different Measurement Points Table S11. Correlational Table of Life Satisfaction between the Different Measurement Points Table S12. Parameters and Model Fit Indices for the Random Intercept Cross‐Lagged Panel Model – SOEP Data Table S13. Correlational Table of Sport Participation between the Different Measurement Points Table S14. Correlational Table of Long‐Standing Health Issues between the Different Measurement Points Table S15. Correlational Table of Self‐Rated Health between the Different Measurement Points Table S16. Correlational Table of Life Satisfaction between the Different Measurement Points Table S17. Parameters and Model Fit Indices for the Random Intercept Cross‐Lagged Panel Model – LISS Data Table S1 [file APHW-17-0-s001.docx]

**Stationarity Constraints**

We tested the tenability of full stationarity constraints (i.e., time-invariant autoregressive and cross-lagged parameters and time-invariant residual variances and covariances) by comparing the fit of a model with the stationarity assumption with the fit of a model without these constraints. We used the Akaike Information Criterion (AIC) and the Bayesian Information Criterion (BIC), as the data were equally spaced in all panels (Mulder & Hamaker, 2021; Preacher & Yaremych, 2023). This assumption means that the underlying processes should be the same regardless of when the measurements were actually taken (e.g., in the interval between T1 and T2 or between T5 and T6). If this assumption is possible, the interpretation of the estimates is facilitated. The AIC was lower in all four panels for the models without constraints. In general, the AIC focuses on efficiency by helping to identify the candidate model that minimizes squared prediction errors or that is expected to cross-validate the best model when applied to new samples (Preacher & Yaremych, 2023). However, models with full stationarity constraints had a lower BIC in three panels (HILDA [Study 1], LISS [Study 3], and UKHLS [Study 4]). For the SOEP (Study 2), the BIC was lower only when residual (co)variances were estimated freely and autoregressive and cross-lagged parameters were constrained to be equal. The BIC emphasizes consistency. If a true model exists and is in the set being compared, the BIC will select the model in the set that lies closest to the true data-generating process (Preacher & Yaremych, 2023). Because the BIC provides an indication that full or partial stationarity constraints are possible, we assumed full stationarity constraints for Studies 1, 3, and 4 and partial stationarity constraints (i.e., time-invariant autoregressive and cross-lagged parameters) for Study 2.

**Missing Data**

As the data were collected in panel studies, missing values are to be expected at both the item and wave levels. Our analyses included all individuals who took part in at least one wave. Full-information maximum likelihood (FIML) estimation was used to handle missing data. However, one assumption of the FIML approach is that the data are missing at least at random (MAR). Data can be considered MAR when the missingness is related to some other variable(s) in the data set but not the missing variable itself (Enders, 2010). Therefore, to improve the FIML approach, we used participants’ age at T1 as an auxiliary variable (Enders, 2008; Graham, 2003). Only adding auxiliary variables that are correlated *r* = .50 or better with the variables of interest will generally help to decrease bias and increase power in the analysis. However, adding auxiliary variables with lower correlations will typically have little incremental benefit, especially when these additional variables are correlated with the auxiliary variables that are already being used. Good candidates for auxiliary variables are the same variables that are used in the analytic model but measured in different waves (Graham, 2009). Since these variables are already included in our longitudinal random intercept model, we decided to include only age in the model at T1 as a possible additional auxiliary variable since the incremental benefit of other potential auxiliary variables seems likely to be small. Because the FIML approach is possible only for continuous variables, we could not include gender as an auxiliary variable. However, within-person coeﬃcient estimates are not aﬀected by time-invariant covariates, such as gender (Usami et al., 2019).

**Study 1: The Household, Income and Labour Dynamics in Australia (HILDA)**

**Method**

***Participants***

We used 22 waves spaced 1 year apart from the HILDA panel (2001 to 2022). HILDA (Watson & Wooden, 2012) is a household-based panel study that collects valuable information about economic and personal well-being, labor market dynamics, and family life in Australia. The average age at T1 was 43.75 years (*SD* = 17.61; *n* = 13,058), and 52.88% of participants at T1 were female. See Table 1 for the age and gender ratios at each time point.

***Measures***

***Physical Activity.*** Physical activity was measured with one item, which was: “In general, how often do you participate in moderate or intensive physical activity for at least 30 minutes?” with the following response options: (1) *not at all*, (2) *less than once a week*, (3) *1 to 2 times a week*, (4) *3 times a week*, (5) *more than 3 times a week*, (6) *every day*. From 2020 on, the question contained the following addition: “Moderate level physical activity will cause a slight increase in breathing and heart rate, such as brisk walking.” Before 2020, no explicit instructions were given with respect to how participants should conceptualize moderate or intensive physical activity.

***Long-Standing Health Issues.*** Participants were asked: “Do you have any long-term health condition, impairment, or disability (such as these) that restricts you in your everyday activities and has lasted or is likely to last for 6 months or more?” with the following response options: (1) *yes*, (2) *no*. Responses to this item were recoded: (0) *yes*, (1) *no*.

***Self-Rated Health.*** Self-rated health was also measured with one item. The item was: “In general, would you say your health is:” with following response options: (1) *excellent*, (2) *very good*, (3) *good*, (4) *fair*, (5) *poor*. Responses to this item were inverted so that a higher score corresponded to a better self-rated health status.

***Life Satisfaction.*** Life satisfaction was also measured with one item. The item was: “All things considered, how satisfied are you with your life?” with response options ranging from (0) *totally dissatisfied* to (10) *totally satisfied*.

**Results**

***Random Intercept Correlations***

We found significant positive correlations between the random intercepts of all variables ($T_{PA\sim\sim LH}$, moderate effect; $T_{PA\sim\sim SH},$ moderate effect; $T_{PA\sim\sim LS},$ small effect; $T_{LH\sim\sim SH}$, large effect; $T_{LH\sim\sim LS},$ small effect; $T_{SH\sim\sim LS},$ moderate effect).

***Autoregressive Effects***

We found significant positive autoregressive effects for all variables ($\gamma_{PA\sim PA}, \gamma_{LH\sim LH}, \gamma_{SH\sim SH},\gamma_{LS\sim LS}$).

***Lagged Effects***

We found small positive bidirectional effects between physical activity and long-standing health issues ($\beta_{LH\sim PA}$; $\beta_{PA\sim LH}$), physical activity and self-rated health ($\beta_{SH\sim PA}$; $\beta_{PA\sim SH}$), and physical activity and life satisfaction ($\beta_{LS\sim PA}$; $\beta_{PA\sim LS}$). Furthermore, we found moderate positive bidirectional effects between long-standing health and self-rated health ($\beta_{LH\sim SH}$; $\beta_{SH\sim LH}$) and small positive bidirectional effects between long-standing health issues and life satisfaction ($\beta_{LH\sim LS}$; $\beta_{LS\sim LH}$) and self-rated health and life satisfaction ($\beta_{SH\sim LS}$; $\beta_{LS\sim SH}$).

**Study 2: German Socio-Economic Panel (SOEP)**

**Method**

***Participants***

We used 14 waves spaced 2 years apart from the SOEP (1995 to 2021). The SOEP (Goebel et al., 2019) is a nationally representative, annual longitudinal panel study of private households and individuals. It is housed in the German Institute for Economic Research (DIW Berlin). The average age at T1 was 43.06 years (*SD* = 16.75; *n* = 13,768), and 51.77% of participants at T1 were female. See Table 1 for the age and gender ratios at each time point.

***Measures***

***Physical Activity.*** Physical activity was measured with one item, which was: “Now some questions about your leisure time. Please indicate how often you take part in sports,” with the following response options: (1) *daily*, (2) *at least once per week*, (3) *at least once per month*, (4) *seldom*, (5) *never*. The responses to this item were inverted so that a higher value corresponded to greater physical activity. Thus, the assessment was based on a broad and subjective understanding of sports and sports activity in leisure time as a measure of physical activity. It should be noted that physical activity is more than only sports participation in leisure time, as it additionally includes occupational activities, conditioning exercises, household tasks (e.g., yardwork, cleaning, and home repair), and other activities (Casperson et al., 1985).

***Long-Standing Health Issues.*** Participants were asked: “Were you on sick leave from work for more than 6 weeks at one time last year?” with following response options: (1) *yes*, (2) *no*. Responses to this item were recoded: (0) *yes*, (1) *no*.

***Self-Rated Health.*** Self-rated health was also measured with one item. The item was: “[How would you describe your current state of health?](https://paneldata.org/question/3e2a1094-0e12-5017-a365-a96a56bf0834)” with following response options: (1) *very good*, (2) *good*, (3) *satisfied*, (4) *less good* , (5) *poor*. Responses to this item were inverted so that a higher score corresponded to a better self-rated health status.

***Life Satisfaction.*** Life satisfaction was also measured with one item. The item was: “How satisfied are you with your life, all things considered?” with response options ranging from (0) *completely dissatisfied* to (10) *completely satisfied*.

**Results**

***Random Intercept Correlations***

We found significant positive correlations between the random intercepts of all variables ($T_{PA\sim\sim LH}$, moderate effect; $T_{PA\sim\sim SH},$ moderate effect; $T_{PA\sim\sim LS},$ moderate effect; $T_{LH\sim\sim SH}$, large effect; $T_{LH\sim\sim LS},$ moderate effect; $T_{SH\sim\sim LS},$ large effect).

***Autoregressive Effects***

We found significant positive autoregressive effects for all variables ($\gamma_{PA\sim PA}, \gamma_{LH\sim LH}, \gamma_{SH\sim SH},\gamma_{LS\sim LS}$).

***Lagged Effects***

We found a small positive effect of physical activity on self-rated health ($\beta_{SH\sim PA}$) and also a small positive reverse effect ($\beta_{PA\sim SH}$). Also, a small positive effect of physical activity on life satisfaction ($\beta_{LS\sim PA}$) and a small positive reverse effect ($\beta_{PA\sim LS}$) were observed. Positive bidirectional effects were found between long-standing health issues and self-rated health ($\beta_{LH\sim SH}$, small effect; $\beta_{SH\sim LH}$, small effect), long-standing health issues and life satisfaction ($\beta_{LH\sim LS}$, small effect; $\beta_{LS\sim LH}, small effect$), and self-rated health and life satisfaction ($\beta_{SH\sim LS}$, small effect; $\beta_{LS\sim SH}$, small effect).

**Study 3: Longitudinal Internet studies for the Social Sciences (LISS) Panel**

**Method**

***Participants***

We used 11 waves spaced 1 year apart from the LISS (2008 to 2018). The LISS panel (Scherpenzeel & Das, 2010) is a representative sample of Dutch individuals including domains such as health, work, education, income, housing, leisure and time use, political views, values, and personality. The average age at T1 was 45.56 years (*SD* = 15.87; *n* = 6,844), and 54.19% of participants at T1 were female. See Table 1 for the age and gender ratios at each time point.

***Measures***

***Light Physical Activity.*** Light physical activity was measured with one item, which was: “If you look back on the last 7 days, on how many of those days did you spend at least 10 minutes walking? Think of walking on the job and at home, walking to get from one place to another, and all the walking you did as part of recreation, sports, or leisure time activities. If you did not walk or walked for less than 10 minutes, enter zero (0).” The possible answers ranged from (0) to (7).

***Moderate Physical Activity.*** Moderate physical activity was measured with one item, which was: “Think of activities that you performed over the last 7 days that required moderate physical exertion. Moderately intensive physical activities cause you to breathe somewhat faster than normally. Again, think only of activities that you performed for at least 10 minutes per occasion. If you think of the past 7 days, on how many of those days did you perform a moderately intensive physical activity, such as carrying light loads, cycling at a normal pace, or a doubles game of tennis? If you did not perform moderately intensive physical activities, enter zero (0).” The possible answers ranged from (0) to (7).

***Vigorous Physical Activity.*** Vigorous physical activity was measured with one item, which was: “If you look back on the last 7 days, on how many of those days did you perform a strenuous physical activity, such as lifting heavy loads, digging, aerobics, or cycling? If you did not perform any strenuous physical activity, enter zero (0).” The possible answers ranged from (0) to (7).

***Long-Standing Health Issues.*** Participants were asked: “Do you suffer from any kind of long-standing disease, affliction, or handicap, or do you suffer from the consequences of an accident?” with following response options: (1) *yes*, (2) *no*. Responses to this item were recoded: (0) *yes*, (1) *no*.

***Self-Rated Health.*** Self-rated health was also measured with one item. The item was: “How would you describe your health, generally speaking?” with the following response options: (1) *poor*, (2) *moderate*, (3) *good*, (4) *very good*, (5) *excellent*.

***Life Satisfaction.*** Life satisfaction was also measured with one item. The item was: “How satisfied are you with the life you lead at the moment?” with response options ranging from (0) *not at all satisfied* to (10) *completely satisfied* (10).

**Results**

***Random Intercept Correlations***

We found significant positive correlations between the random intercepts of all variables ($T_{LPA\sim\sim MPA}$, moderate effect; $T_{LPA\sim\sim VPA},$ small effect; $T_{LPA\sim\sim LH},$ small effect; $T_{LPA\sim\sim SH},$ small effect; $T_{LPA\sim\sim LS},$ small effect; $T_{MPA\sim\sim VPA}$, moderate effect; $T_{MPA\sim\sim LH},$ small effect; $T_{MPA\sim\sim SH},$ small effect; $T_{MPA\sim\sim LS},$ small effect; $T_{VPA\sim\sim LH},$ small effect; $T_{VPA\sim\sim SH},$ small effect; $T_{VPA\sim\sim LS},$ small effect; $T_{LH\sim\sim SH},$ large effect; $T_{LH\sim\sim LS},$ small effect; $T_{SH\sim\sim LS},$ moderate effect).

***Autoregressive Effects***

We found significant positive autoregressive effects for all variables ($\gamma_{LPA\sim LPA}, \gamma_{MPA\sim MPA},\gamma_{VPA\sim VPA},\gamma_{LH\sim LH}, \gamma_{SH\sim SH},\gamma_{LS\sim LS}$).

***Lagged Effects***

We found a small positive effect of light physical activity on moderate physical activity ($\beta_{MPA\sim LPA}$). Also, a small positive effect of moderate physical activity on vigorous physical activity ($\beta_{VPA\sim MPA}$) and a small positive reverse effect ($\beta_{MPA\sim VPA}$) were observed. Additionally, a small negative effect of vigorous physical activity on long-standing health issues ($\beta_{LH\sim VPA}$) was found. There were positive bidirectional effects between vigorous physical activity and self-rated health ($\beta_{SH\sim VPA}$, small effect; $\beta_{VPA\sim SH}, small effect$). Also, a positive small effect of moderate physical activity on self-rated health emerged $(\beta_{SH\sim MPA}$). Moreover, a positive small effect of moderate physical activity on life satisfaction ($\beta_{LS\sim MPA}$) was found. Positive bidirectional effects were found between long-standing health issues and self-rated health ($\beta_{LH\sim SH}$, small effect; $\beta_{SH\sim LH}$, small effect), between long-standing health issues and life satisfaction ($\beta_{LH\sim LS}$, small effect; $\beta_{LS\sim LH}, small effect$), and between self-rated health and life satisfaction ($\beta_{SH\sim LS}$, moderate effect; $\beta_{LS\sim SH}$, small effect).

**Study 4: The UK (Understanding Society) Household Longitudinal Study (UKHLS)**

**Method**

***Participants***

We used four waves spaced 2 years apart from the UKHLS (2015/16 to 2021/22). The UKHLS (University of Essex, 2023) is a panel survey of United Kingdom households. The average age at T1 was 48.07 years (*SD* = 18.70; *n* = 42,141), and 53.94% of participants at T1 were female. See Table 1 for the age and gender ratios at each time point.

***Measures***

***Light Physical Activity.*** Light physical activity was measured with one item, which was: “Now think about the time you spent walking in the last 7 days. This includes at work and at home, walking to travel from place to place, and any other walking that you might do solely for recreation, sport, exercise, or leisure. During the last 7 days, on how many days did you walk for at least 10 minutes at a time?” The possible answers ranged from (0) to (7).

***Moderate Physical Activity.*** Moderate physical activity was measured with one item, which was: “Now think about activities that take moderate physical effort that you did in the last 7 days. Moderate physical activities make you breathe somewhat harder than normal and may include carrying light loads, bicycling at a regular pace, or doubles tennis. Do not include walking. Again, think only about those physical activities that you did for at least 10 minutes at a time. In the last 7 days, on how many days did you engage in moderate physical activities?” The possible answers ranged from (0) to (7).

***Vigorous Physical Activity.*** Vigorous physical activity was measured with one item, which was: “Now, think about all the vigorous activities that take hard physical effort that you did in the last 7 days. Vigorous activities make you breathe much harder than normal and may include heavy lifting, digging, aerobics, or fast bicycling. Think only about those physical activities that you did for at least 10 minutes at a time. During the last 7 days, on how many days did you engage in vigorous physical activities?” The possible answers ranged from (0) to (7).

***Long-Standing Health Issues.*** Participants were asked: “Do you have any long-standing physical or mental impairment, illness, or disability? By 'long-standing' I mean anything that has troubled you over a period of at least 12 months or that is likely to trouble you over a period of at least 12 months.” The item was answered with following response options: (1) *yes*, (2) *no*. Responses to this item were recoded: (0) *yes*, (1) *no*.

***Self-Rated Health.*** Self-rated health was also measured with one item. The item was: “In general, would you say your health is...” with the following response options: (1) *excellent*, (2) *very good*, (3) *good*, (4) *fair*, (5) *poor*. Responses to this item were inverted so that a higher score corresponded to a better self-rated health status.

***Life Satisfaction.*** Life satisfaction was also measured with one item. The item was: “Here are some questions about how you feel about your life. Please choose the number which you feel best describes how dissatisfied or satisfied you are with the following aspects of your current situation: Your life overall.” The item was answered with the following response options: (1) *completely dissatisfied*, (2) *mostly dissatisfied*, (3) *somewhat dissatisfied*, (4) *neither sat nor dissat*, (5) *somewhat satisfied*, (6) *mostly satisfied*, (7) *completely satisfied*.

**Results**

***Random Intercept Correlations***

We found significant positive correlations between the random intercepts of all variables ($T_{LPA\sim\sim MPA}$, large effect; $T_{LPA\sim\sim VPA},$ moderate effect; $T_{LPA\sim\sim LH},$ moderate effect; $T_{LPA\sim\sim SH},$ moderate effect; $T_{LPA\sim\sim LS},$ small effect; $T_{MPA\sim\sim VPA}$, large effect; $T_{MPA\sim\sim LH},$ small effect; $T_{MPA\sim\sim SH},$ moderate effect; $T_{MPA\sim\sim LS},$ small effect; $T_{VPA\sim\sim LH},$ moderate effect; $T_{VPA\sim\sim SH},$ moderate effect; $T_{VPA\sim\sim LS},$ small effect; $T_{LH\sim\sim SH},$ large effect; $T_{LH\sim\sim LS},$ moderate effect; $T_{SH\sim\sim LS},$ large effect).

***Autoregressive Effects***

We found significant positive autoregressive effects for all variables ($\gamma_{LPA\sim LPA}, \gamma_{MPA\sim MPA},\gamma_{VPA\sim VPA},\gamma_{LH\sim LH}, \gamma_{SH\sim SH},\gamma_{LS\sim LS}$).

***Lagged Effects***

We found positive bidirectional effects between light physical activity and moderate physical activity ($\beta_{MPA\sim LPA}$, small effect; $\beta_{LPA\sim MPA}, small effect$), light physical activity and vigorous physical activity ($\beta_{VPA\sim LPA}$, small effect; $\beta_{LPA\sim VPA}, small effect$), and moderate physical activity and vigorous physical activity ($\beta_{VPA\sim MPA}$, small effect; $\beta_{MPA\sim VPA}, small effect$). Additionally, a small positive effect of long-standing health issues on vigorous physical activity ($\beta_{VPA\sim LH}$) was found. Moreover, we found positive bidirectional effects between light physical activity and self-rated health ($\beta_{SH\sim LPA}$, small effect; $\beta_{LPA\sim SH}, small effect$), moderate physical activity and self-rated health ($\beta_{SH\sim MPA}$, small effect; $\beta_{MPA\sim SH}, small effect$), and vigorous physical activity and self-rated health ($\beta_{SH\sim VPA}$, small effect; $\beta_{VPA\sim SH}, small effect$). Furthermore, a bidirectional effect between light physical activity and life satisfaction was found ($\beta_{LS\sim LPA}$, small effect; $\beta_{LPA\sim LS}, small effect$). Positive bidirectional effects were found between long-standing health issues and self-rated health ($\beta_{LH\sim SH}$, small effect; $\beta_{SH\sim LH}$, small effect), long-standing health issues and life satisfaction ($\beta_{LH\sim LS}$, small effect; $\beta_{LS\sim LH}, small effect$), and self-rated health and life satisfaction ($\beta_{SH\sim LS}$, small effect; $\beta_{LS\sim SH}$, small effect).

| **Table 1**  *Characteristics of the Four Panel Studies* | | |
| --- | --- | --- |
| Intensity of physical activity | Time interval between waves | |
|  | 1-year interval | 2-year interval |
| Moderate-to-vigorous physical activity | Study 1: HILDA  21 waves (2001 to 2021)  *N* = 32,913  *M*_age_ T1 = 43.75 years (*SD* = 17.61; *n* = 13,058)  50.88% were women (*n* = 13,058) at T1 | Study 2: SOEP  14 waves (1995 to 2021)  *N* = 83,844  *M*_age_ T1 = 43.06 years (*SD* = 16.75; *n* = 13,768)  50.77% were women (*n* = 13,768) at T1 |
| Varying physical activities (i.e., light, moderate, vigorous) | Study 3: LISS  11 waves (2008 to 2018)  *N* = 14,778  *M*_age_ T1 = 45.56 years (*SD* = 15.87; *n* = 6,844)  55.19% were women (*n* = 6,878) at T1 | Study 4: UKHLS  4 waves (2015/16 to 2021/22)  *N* = 50,032  *M*_age_ T1 = 48.07 years (*SD* = 18.70; *n* = 42,141)  53.94% were women (*n* = 42,143) at T1 |

| **Table 2**  *Age and Gender Ratios at Each Time Point* | | | | | |
| --- | --- | --- | --- | --- | --- |
|  |  | Study 1: HILDA | Study 2: SOEP | Study 3: LISS | Study 4: UKHLS |
| T1 | Age (*SD*)  Range  *n* | 43.75 (17.61)  15 – 100  13,058 | 43.06 (16.75)  16 – 98  13,768 | 45.56 (15.87)  15 – 108  6,844 | 48.07 (18.70)  15 – 101  42,141 |
|  | Gender (female)  *n* | 52.88%  13,058 | 51.77%  13,768 | 54.19%  6,878 | 53.94%  42,143 |
| T2 | Age (*SD*)  Range  *n* | 44.07 (17.68)  15 – 97  12,130 | 43.60 (16.86)  17 – 98  13,283 | 46.52 (16.50)  16 – 95  5,572 | 49.34 (18.81)  16 – 103  36,039 |
|  | Gender (female)  *n* | 52.72%  12,130 | 51.67%  13,283 | 54.11%  5,572 | 55.00%  36,039 |
| T3 | Age (*SD*)  Range  *n* | 44.21 (17.78)  15 – 98  11,747 | 44.46 (17.03)  16 – 98  14,085 | 48.52 (17.22)  16 – 96  5,827 | 50.26 (18.84)  16 – 100  31,984 |
|  | Gender (female)  *n* | 52,99%  11,747 | 51.68%  14,085 | 53.77  5,827 | 55.44  31,984 |
| T4 | Age (*SD*)  Range  *n* | 44.47 (17.96)  15 – 99  11,397 | 46.17 (17.30)  17 – 99  22,351 | 49.35 (17.36)  16 – 97  5,316 | 50.96 (18.65)  16 – 101  27,968 |
|  | Gender (female)  *n* | 53,20%  11,397 | 52.03%  22,351 | 53.67%  5,316 | 55.69%  27,969 |
| T5 | Age (*SD*)  Range  *n* | 44.48 (18.20)  15 – 100  11,465 | 46.89 (17.35)  16 – 100  22,611 | 49.63 (17.34)  16 – 94  5,892 |  |
|  | Gender (female)  *n* | 53.41%  11,465 | 51.87%  22,611 | 53.90%  5,892 |  |
| T6 | Age (*SD*)  Range  *n* | 44.50 (18.40)  15 – 96  11,688 | 47.42 (17.59)  16 – 96  21,105 | 50.17 (17.55)  16 – 92  5,612 |  |
|  | Gender (female)  *n* | 53.39%  11,688 | 52.18%  22,105 | 53.72%  5,612 |  |
| T7 | Age (*SD*)  Range  *n* | 44.81 (18.54)  15 – 97  11,381 | 48.98 (17.52)  18 – 98  20,886 | 49.70 (17.82)  16 – 96  6,294 |  |
|  | Gender (female)  *n* | 53.62%  11,381 | 52.41%  20,886 | 54.18%  6,294 |  |
| T8 | Age (*SD*)  Range  *n* | 44.94 (18.65)  15 – 96  11,193 | 49.91 (17.71)  17 – 100  20,792 | 50.46 (17.99)  15 – 97  5,959 |  |
|  | Gender (female)  *n* | 53.61%  11,193 | 52.31%  20,792 | 53.90%  5,959 |  |
| T9 | Age (*SD*)  Range  *n* | 44.82 (18.64)  15 – 98  11,563 | 47.70 (16.94)  16 – 101  28,733 | 51.75 (18.01)  16 – 98  5,252 |  |
|  | Gender (female)  *n* | 53.31%  11,563 | 54.71%  28,733 | 52.88%  5,252 |  |
| T10 | Age (*SD*)  Range  *n* | 44.72 (18.74)  15 – 98  12,052 | 46.97 (17.06)  16 – 103  30,956 | 50.97 (18.15)  16 – 100  6,008 |  |
|  | Gender (female)  *n* | 53.09%  12,052 | 54.36%  30,956 | 54.61%  6,008 |  |
| T11 | Age (*SD*)  Range  *n* | 45.07 (18.69)  15 – 99  15,366 | 47.75 (17.48)  17 – 105  27,183 | 51.95 (18.23)  16 – 101  5,378 |  |
|  | Gender (female)  *n* | 53.00%  15,366 | 54.02%  27,183 | 54.37%  5,378 |  |
| T12 | Age (*SD*)  Range  *n* | 45.22 (18.76)  15 – 100  15,389 | 45.97 (17.49)  16 – 102  32,485 |  |  |
|  | Gender (female)  *n* | 53.81%  15,389 | 51.41%  32,485 |  |  |
| T13 | Age (*SD*)  Range  *n* | 45.23 (18.82)  15 – 101  15,360 | 48.14 (17.38)  15 – 102  29,905 |  |  |
|  | Gender (female)  *n* | 53.35%  15,360 | 50.62%  29,905 |  |  |
| T14 | Age (*SD*)  Range  *n* | 45.37 (18.94)  15 – 98  15,595 | 48.23 (17.40)  18 – 102  20,561 |  |  |
|  | Gender (female)  *n* | 53.14%  15,595 | 51.36  20,561 |  |  |
| T15 | Age (*SD*)  Range  *n* | 45.76 (19.05)  15 – 99  15,513 |  |  |  |
|  | Gender (female)  *n* | 53.17%  15,513 |  |  |  |
| T16 | Age (*SD*)  Range  *n* | 45.57 (19.04)  15 -99  16,253 |  |  |  |
|  | Gender (female)  *n* | 52.97%  16,253 |  |  |  |
| T17 | Age (*SD*)  Range  *n* | 45.90 (19.06)  15 – 100  16,140 |  |  |  |
|  | Gender (female)  *n* | 53.10  16,140 |  |  |  |
| T18 | Age (*SD*)  Range  *n* | 46.25 (19.11)  15 – 100  15,887 |  |  |  |
|  | Gender (female)  *n* | 53.02%  15,887 |  |  |  |
| T19 | Age (*SD*)  Range  *n* | 46.38 (19.24)  15 – 101  16,082 |  |  |  |
|  | Gender (female)  *n* | 53.03%  16,082 |  |  |  |
| T20 | Age (*SD*)  Range  *n* | 46.61 (19.16)  15 – 99  15,676 |  |  |  |
|  | Gender (female)  *n* | 53.89%  15,676 |  |  |  |
| T21 | Age (*SD*)  Range  *n* | 46.90 (19.25)  15 – 100  15,299 |  |  |  |
|  | Gender (female)  *n* | 53.83%  15,299 |  |  |  |
| T22 | Age (*SD*)  Range  *n* | 47.47 (19.36)  15 – 101  14,814 |  |  |  |
|  | Gender (female)  *n* | 53.94%  14,814 |  |  |  |
|  | | | | | |

| **Table 3**  *Descriptives and Distributions for Physical Activity, Long-Standing Health Issues, Subjective Health, and Life Satisfaction at All Time Points – HILDA Data* | | | | | | | | | | | |
| --- | --- | --- | --- | --- | --- | --- | --- | --- | --- | --- | --- |
| Variable | Wave | n_Missing | Complete Rate | *M* | *SD* | 0th Percentile | 25th Percentile | 50th Percentile | 75th Percentile | | 100th Percentile |
| PA | 1 | 85 | 0.99 | 3.60 | 1.59 | 1 | 2 | 4 | 5 | 6 | |
| PA | 2 | 62 | 0.99 | 3.58 | 1.57 | 1 | 2 | 3 | 5 | 6 | |
| PA | 3 | 67 | 0.99 | 3.56 | 1.57 | 1 | 2 | 3 | 5 | 6 | |
| PA | 4 | 78 | 0.99 | 3.57 | 1.55 | 1 | 2 | 3 | 5 | 6 | |
| PA | 5 | 60 | 0.99 | 3.59 | 1.55 | 1 | 2 | 3 | 5 | 6 | |
| PA | 6 | 50 | 1.00 | 3.61 | 1.54 | 1 | 2 | 4 | 5 | 6 | |
| PA | 7 | 65 | 0.99 | 3.64 | 1.53 | 1 | 2 | 4 | 5 | 6 | |
| PA | 8 | 139 | 0.99 | 3.59 | 1.53 | 1 | 2 | 3 | 5 | 6 | |
| PA | 9 | 121 | 0.99 | 3.64 | 1.54 | 1 | 2 | 4 | 5 | 6 | |
| PA | 10 | 62 | 0.99 | 3.56 | 1.54 | 1 | 2 | 3 | 5 | 6 | |
| PA | 11 | 58 | 1.00 | 3.56 | 1.55 | 1 | 2 | 3 | 5 | 6 | |
| PA | 12 | 59 | 1.00 | 3.57 | 1.55 | 1 | 2 | 3 | 5 | 6 | |
| PA | 13 | 68 | 1.00 | 3.62 | 1.55 | 1 | 2 | 4 | 5 | 6 | |
| PA | 14 | 49 | 1.00 | 3.55 | 1.55 | 1 | 2 | 3 | 5 | 6 | |
| PA | 15 | 104 | 0.99 | 3.53 | 1.56 | 1 | 2 | 3 | 5 | 6 | |
| PA | 16 | 52 | 1.00 | 3.52 | 1.56 | 1 | 2 | 3 | 5 | 6 | |
| PA | 17 | 159 | 0.99 | 3.53 | 1.57 | 1 | 2 | 3 | 5 | 6 | |
| PA | 18 | 105 | 0.99 | 3.53 | 1.56 | 1 | 2 | 3 | 5 | 6 | |
| PA | 19 | 91 | 0.99 | 3.54 | 1.57 | 1 | 2 | 3 | 5 | 6 | |
| PA | 20 | 109 | 0.99 | 3.57 | 1.58 | 1 | 2 | 3 | 5 | 6 | |
| PA | 21 | 83 | 0.99 | 3.57 | 1.57 | 1 | 2 | 3 | 5 | 6 | |
| PA | 22 | 70 | 1.00 | 3.50 | 1.56 | 1 | 2 | 3 | 5 | 6 | |
| LH | 1 | 0 | 1.00 | 0.77 | 0.42 | 0 | 1 | 1 | 1 | 1 | |
| LH | 2 | 0 | 1.00 | 0.78 | 0.41 | 0 | 1 | 1 | 1 | 1 | |
| LH | 3 | 0 | 1.00 | 0.72 | 0.45 | 0 | 0 | 1 | 1 | 1 | |
| LH | 4 | 0 | 1.00 | 0.74 | 0.44 | 0 | 0 | 1 | 1 | 1 | |
| LH | 5 | 0 | 1.00 | 0.72 | 0.45 | 0 | 0 | 1 | 1 | 1 | |
| LH | 6 | 0 | 1.00 | 0.73 | 0.44 | 0 | 0 | 1 | 1 | 1 | |
| LH | 7 | 1 | 1.00 | 0.73 | 0.45 | 0 | 0 | 1 | 1 | 1 | |
| LH | 8 | 0 | 1.00 | 0.74 | 0.44 | 0 | 0 | 1 | 1 | 1 | |
| LH | 9 | 6 | 1.00 | 0.72 | 0.45 | 0 | 0 | 1 | 1 | 1 | |
| LH | 10 | 3 | 1.00 | 0.73 | 0.44 | 0 | 0 | 1 | 1 | 1 | |
| LH | 11 | 2 | 1.00 | 0.73 | 0.45 | 0 | 0 | 1 | 1 | 1 | |
| LH | 12 | 4 | 1.00 | 0.73 | 0.44 | 0 | 0 | 1 | 1 | 1 | |
| LH | 13 | 3 | 1.00 | 0.70 | 0.46 | 0 | 0 | 1 | 1 | 1 | |
| LH | 14 | 3 | 1.00 | 0.71 | 0.45 | 0 | 0 | 1 | 1 | 1 | |
| LH | 15 | 1 | 1.00 | 0.71 | 0.45 | 0 | 0 | 1 | 1 | 1 | |
| LH | 16 | 1 | 1.00 | 0.72 | 0.45 | 0 | 0 | 1 | 1 | 1 | |
| LH | 17 | 6 | 1.00 | 0.70 | 0.46 | 0 | 0 | 1 | 1 | 1 | |
| LH | 18 | 6 | 1.00 | 0.71 | 0.46 | 0 | 0 | 1 | 1 | 1 | |
| LH | 19 | 1 | 1.00 | 0.70 | 0.46 | 0 | 0 | 1 | 1 | 1 | |
| LH | 20 | 4 | 1.00 | 0.70 | 0.46 | 0 | 0 | 1 | 1 | 1 | |
| LH | 21 | 15 | 1.00 | 0.69 | 0.46 | 0 | 0 | 1 | 1 | 1 | |
| LH | 22 | 7 | 1.00 | 0.68 | 0.47 | 0 | 0 | 1 | 1 | 1 | |
| SH | 1 | 141 | 0.99 | 3.48 | 1.01 | 1 | 3 | 4 | 4 | 5 | |
| SH | 2 | 325 | 0.97 | 3.40 | 0.97 | 1 | 3 | 3 | 4 | 5 | |
| SH | 3 | 237 | 0.98 | 3.38 | 0.98 | 1 | 3 | 3 | 4 | 5 | |
| SH | 4 | 80 | 0.99 | 3.36 | 0.97 | 1 | 3 | 3 | 4 | 5 | |
| SH | 5 | 137 | 0.99 | 3.35 | 0.96 | 1 | 3 | 3 | 4 | 5 | |
| SH | 6 | 381 | 0.97 | 3.38 | 0.97 | 1 | 3 | 3 | 4 | 5 | |
| SH | 7 | 201 | 0.98 | 3.38 | 0.96 | 1 | 3 | 3 | 4 | 5 | |
| SH | 8 | 85 | 0.99 | 3.37 | 0.95 | 1 | 3 | 3 | 4 | 5 | |
| SH | 9 | 237 | 0.98 | 3.45 | 0.97 | 1 | 3 | 4 | 4 | 5 | |
| SH | 10 | 94 | 0.99 | 3.37 | 0.96 | 1 | 3 | 3 | 4 | 5 | |
| SH | 11 | 135 | 0.99 | 3.40 | 0.97 | 1 | 3 | 3 | 4 | 5 | |
| SH | 12 | 102 | 0.99 | 3.40 | 0.96 | 1 | 3 | 3 | 4 | 5 | |
| SH | 13 | 105 | 0.99 | 3.40 | 0.97 | 1 | 3 | 3 | 4 | 5 | |
| SH | 14 | 157 | 0.99 | 3.36 | 0.97 | 1 | 3 | 3 | 4 | 5 | |
| SH | 15 | 187 | 0.99 | 3.35 | 0.97 | 1 | 3 | 3 | 4 | 5 | |
| SH | 16 | 106 | 0.99 | 3.37 | 0.96 | 1 | 3 | 3 | 4 | 5 | |
| SH | 17 | 210 | 0.99 | 3.36 | 0.97 | 1 | 3 | 3 | 4 | 5 | |
| SH | 18 | 314 | 0.98 | 3.34 | 0.97 | 1 | 3 | 3 | 4 | 5 | |
| SH | 19 | 111 | 0.99 | 3.33 | 0.97 | 1 | 3 | 3 | 4 | 5 | |
| SH | 20 | 58 | 1.00 | 3.40 | 0.95 | 1 | 3 | 3 | 4 | 5 | |
| SH | 21 | 153 | 0.99 | 3.39 | 0.96 | 1 | 3 | 3 | 4 | 5 | |
| SH | 22 | 88 | 0.99 | 3.32 | 0.96 | 1 | 3 | 3 | 4 | 5 | |
| LS | 1 | 13 | 1.00 | 7.99 | 1.65 | 0 | 7 | 8 | 9 | 10 | |
| LS | 2 | 3 | 1.00 | 7.91 | 1.57 | 0 | 7 | 8 | 9 | 10 | |
| LS | 3 | 2 | 1.00 | 8.00 | 1.51 | 0 | 7 | 8 | 9 | 10 | |
| LS | 4 | 7 | 1.00 | 7.97 | 1.51 | 0 | 7 | 8 | 9 | 10 | |
| LS | 5 | 3 | 1.00 | 7.92 | 1.46 | 0 | 7 | 8 | 9 | 10 | |
| LS | 6 | 3 | 1.00 | 7.91 | 1.46 | 0 | 7 | 8 | 9 | 10 | |
| LS | 7 | 1 | 1.00 | 7.93 | 1.43 | 0 | 7 | 8 | 9 | 10 | |
| LS | 8 | 12 | 1.00 | 7.92 | 1.39 | 0 | 7 | 8 | 9 | 10 | |
| LS | 9 | 10 | 1.00 | 7.92 | 1.41 | 0 | 7 | 8 | 9 | 10 | |
| LS | 10 | 3 | 1.00 | 7.90 | 1.42 | 0 | 7 | 8 | 9 | 10 | |
| LS | 11 | 4 | 1.00 | 7.95 | 1.44 | 0 | 7 | 8 | 9 | 10 | |
| LS | 12 | 8 | 1.00 | 7.94 | 1.42 | 0 | 7 | 8 | 9 | 10 | |
| LS | 13 | 4 | 1.00 | 7.93 | 1.42 | 0 | 7 | 8 | 9 | 10 | |
| LS | 14 | 4 | 1.00 | 7.93 | 1.43 | 0 | 7 | 8 | 9 | 10 | |
| LS | 15 | 3 | 1.00 | 7.95 | 1.45 | 0 | 7 | 8 | 9 | 10 | |
| LS | 16 | 1 | 1.00 | 7.94 | 1.43 | 0 | 7 | 8 | 9 | 10 | |
| LS | 17 | 8 | 1.00 | 7.92 | 1.45 | 0 | 7 | 8 | 9 | 10 | |
| LS | 18 | 9 | 1.00 | 7.97 | 1.42 | 0 | 7 | 8 | 9 | 10 | |
| LS | 19 | 8 | 1.00 | 7.97 | 1.43 | 0 | 7 | 8 | 9 | 10 | |
| LS | 20 | 10 | 1.00 | 7.99 | 1.38 | 0 | 7 | 8 | 9 | 10 | |
| LS | 21 | 9 | 1.00 | 7.97 | 1.38 | 0 | 7 | 8 | 9 | 10 | |
| LS | 22 | 5 | 1.00 | 8.00 | 1.36 | 0 | 7 | 8 | 9 | 10 | |
| *Note.* PA = moderate-to-vigorous physical activity; LH = long-standing health issues; SH = self-rated health; LS = life satisfaction. | | | | | | | | | | | |

| **Table 4**  *Descriptives and Distributions for Physical Activity, Long-Standing Health Issues, Subjective Health, and Life Satisfaction at All Time Points – SOEP Data* | | | | | | | | | | |
| --- | --- | --- | --- | --- | --- | --- | --- | --- | --- | --- |
| Variable | Wave | n_Missing | Complete Rate | *M* | *SD* | 0th Percentile | 25th Percentile | 50th Percentile | 75th Percentile | 100th Percentile |
| PA | 1 | 103 | 0.99 | 2.20 | 1.33 | 1 | 1 | 2 | 4 | 5 |
| PA | 2 | 143 | 0.99 | 2.00 | 1.22 | 1 | 1 | 1 | 3 | 4 |
| PA | 3 | 136 | 0.99 | 2.06 | 1.23 | 1 | 1 | 2 | 3 | 4 |
| PA | 4 | 206 | 0.99 | 2.10 | 1.28 | 1 | 1 | 2 | 4 | 4 |
| PA | 5 | 140 | 0.99 | 2.48 | 1.41 | 1 | 1 | 2 | 4 | 5 |
| PA | 6 | 141 | 0.99 | 2.33 | 1.30 | 1 | 1 | 2 | 4 | 4 |
| PA | 7 | 127 | 0.99 | 2.37 | 1.32 | 1 | 1 | 2 | 4 | 4 |
| PA | 8 | 100 | 1.00 | 2.42 | 1.32 | 1 | 1 | 2 | 4 | 4 |
| PA | 9 | 116 | 1.00 | 2.39 | 1.34 | 1 | 1 | 2 | 4 | 4 |
| PA | 10 | 5262 | 0.83 | 2.80 | 1.52 | 1 | 1 | 3 | 4 | 5 |
| PA | 11 | 102 | 1.00 | 2.51 | 1.37 | 1 | 1 | 2 | 4 | 4 |
| PA | 12 | 104 | 1.00 | 2.54 | 1.40 | 1 | 1 | 2 | 4 | 5 |
| PA | 13 | 357 | 0.99 | 3.00 | 1.54 | 1 | 1 | 4 | 4 | 5 |
| PA | 14 | 2607 | 0.87 | 3.19 | 1.39 | 1 | 2 | 4 | 4 | 5 |
| LH | 1 | 9646 | 0.30 | 0.90 | 0.30 | 0 | 1 | 1 | 1 | 1 |
| LH | 2 | 5258 | 0.60 | 0.94 | 0.24 | 0 | 1 | 1 | 1 | 1 |
| LH | 3 | 4933 | 0.65 | 0.94 | 0.23 | 0 | 1 | 1 | 1 | 1 |
| LH | 4 | 8384 | 0.62 | 0.94 | 0.23 | 0 | 1 | 1 | 1 | 1 |
| LH | 5 | 8465 | 0.63 | 0.95 | 0.22 | 0 | 1 | 1 | 1 | 1 |
| LH | 6 | 8278 | 0.61 | 0.96 | 0.21 | 0 | 1 | 1 | 1 | 1 |
| LH | 7 | 8298 | 0.60 | 0.96 | 0.20 | 0 | 1 | 1 | 1 | 1 |
| LH | 8 | 8232 | 0.60 | 0.95 | 0.22 | 0 | 1 | 1 | 1 | 1 |
| LH | 9 | 10681 | 0.63 | 0.95 | 0.23 | 0 | 1 | 1 | 1 | 1 |
| LH | 10 | 10773 | 0.65 | 0.94 | 0.23 | 0 | 1 | 1 | 1 | 1 |
| LH | 11 | 9497 | 0.65 | 0.95 | 0.22 | 0 | 1 | 1 | 1 | 1 |
| LH | 12 | 14657 | 0.55 | 0.94 | 0.24 | 0 | 1 | 1 | 1 | 1 |
| LH | 13 | 12044 | 0.60 | 0.94 | 0.24 | 0 | 1 | 1 | 1 | 1 |
| LH | 14 | 6443 | 0.69 | 0.95 | 0.23 | 0 | 1 | 1 | 1 | 1 |
| SH | 1 | 42 | 1.00 | 3.41 | 0.96 | 1 | 3 | 4 | 4 | 5 |
| SH | 2 | 17 | 1.00 | 3.41 | 0.94 | 1 | 3 | 4 | 4 | 5 |
| SH | 3 | 27 | 1.00 | 3.41 | 0.95 | 1 | 3 | 4 | 4 | 5 |
| SH | 4 | 27 | 1.00 | 3.42 | 0.97 | 1 | 3 | 4 | 4 | 5 |
| SH | 5 | 38 | 1.00 | 3.42 | 0.96 | 1 | 3 | 4 | 4 | 5 |
| SH | 6 | 69 | 1.00 | 3.38 | 0.96 | 1 | 3 | 3 | 4 | 5 |
| SH | 7 | 53 | 1.00 | 3.36 | 0.95 | 1 | 3 | 3 | 4 | 5 |
| SH | 8 | 43 | 1.00 | 3.35 | 0.96 | 1 | 3 | 3 | 4 | 5 |
| SH | 9 | 27 | 1.00 | 3.41 | 0.98 | 1 | 3 | 4 | 4 | 5 |
| SH | 10 | 20 | 1.00 | 3.44 | 0.99 | 1 | 3 | 4 | 4 | 5 |
| SH | 11 | 36 | 1.00 | 3.42 | 0.97 | 1 | 3 | 4 | 4 | 5 |
| SH | 12 | 40 | 1.00 | 3.50 | 1.01 | 1 | 3 | 4 | 4 | 5 |
| SH | 13 | 23 | 1.00 | 3.51 | 0.99 | 1 | 3 | 4 | 4 | 5 |
| SH | 14 | 19 | 1.00 | 3.52 | 1.02 | 1 | 3 | 4 | 4 | 5 |
| LS | 1 | 70 | 0.99 | 6.89 | 1.83 | 0 | 6 | 7 | 8 | 10 |
| LS | 2 | 24 | 1.00 | 6.79 | 1.79 | 0 | 6 | 7 | 8 | 10 |
| LS | 3 | 28 | 1.00 | 6.97 | 1.78 | 0 | 6 | 7 | 8 | 10 |
| LS | 4 | 48 | 1.00 | 7.10 | 1.74 | 0 | 6 | 7 | 8 | 10 |
| LS | 5 | 42 | 1.00 | 6.96 | 1.78 | 0 | 6 | 7 | 8 | 10 |
| LS | 6 | 64 | 1.00 | 6.95 | 1.83 | 0 | 6 | 7 | 8 | 10 |
| LS | 7 | 52 | 1.00 | 6.95 | 1.78 | 0 | 6 | 7 | 8 | 10 |
| LS | 8 | 57 | 1.00 | 6.98 | 1.78 | 0 | 6 | 7 | 8 | 10 |
| LS | 9 | 2601 | 0.91 | 7.18 | 1.74 | 0 | 6 | 8 | 8 | 10 |
| LS | 10 | 71 | 1.00 | 7.31 | 1.75 | 0 | 7 | 8 | 8 | 10 |
| LS | 11 | 62 | 1.00 | 7.38 | 1.73 | 0 | 7 | 8 | 8 | 10 |
| LS | 12 | 63 | 1.00 | 7.34 | 1.77 | 0 | 7 | 8 | 8 | 10 |
| LS | 13 | 80 | 1.00 | 7.54 | 1.71 | 0 | 7 | 8 | 9 | 10 |
| LS | 14 | 99 | 1.00 | 7.51 | 1.72 | 0 | 7 | 8 | 9 | 10 |
| *Note.* PA = sport participation in leisure time; LH = long-standing health issues; SH = self-rated health; LS = life satisfaction. | | | | | | | | | | |

| **Table 5**  *Descriptives and Distributions for Light Physical Activity, Moderate Physical Activity, Vigorous Physical Activity, Long-Standing Health Issues, Subjective Health, and Life Satisfaction at All Time Points – LISS Data* | | | | | | | | | | |
| --- | --- | --- | --- | --- | --- | --- | --- | --- | --- | --- |
| Variable | Wave | n_Missing | Complete Rate | *M* | *SD* | 0th Percentile | 25th Percentile | 50th Percentile | 75th Percentile | 100th Percentile |
| LPA | 1 | 1019 | 0.87 | 4.54 | 2.53 | 0 | 2 | 5 | 7 | 7 |
| LPA | 2 | 480 | 0.93 | 4.26 | 2.60 | 0 | 2 | 5 | 7 | 7 |
| LPA | 3 | 850 | 0.88 | 4.12 | 2.64 | 0 | 2 | 5 | 7 | 7 |
| LPA | 4 | 382 | 0.94 | 4.05 | 2.66 | 0 | 2 | 5 | 7 | 7 |
| LPA | 5 | 1446 | 0.78 | 4.13 | 2.66 | 0 | 2 | 5 | 7 | 7 |
| LPA | 6 | 380 | 0.94 | 3.99 | 2.68 | 0 | 2 | 4 | 7 | 7 |
| LPA | 7 | 1810 | 0.75 | 3.87 | 2.67 | 0 | 1 | 4 | 7 | 7 |
| LPA | 8 | 488 | 0.92 | 4.09 | 2.67 | 0 | 2 | 5 | 7 | 7 |
| LPA | 9 | 1399 | 0.79 | 4.03 | 2.65 | 0 | 2 | 5 | 7 | 7 |
| LPA | 10 | 518 | 0.92 | 4.11 | 2.62 | 0 | 2 | 5 | 7 | 7 |
| LPA | 11 | 360 | 0.94 | 4.14 | 2.62 | 0 | 2 | 5 | 7 | 7 |
| MPA | 1 | 1018 | 0.87 | 3.16 | 2.51 | 0 | 1 | 3 | 5 | 7 |
| MPA | 2 | 477 | 0.93 | 2.95 | 2.52 | 0 | 0 | 3 | 5 | 7 |
| MPA | 3 | 847 | 0.88 | 2.90 | 2.56 | 0 | 0 | 2 | 5 | 7 |
| MPA | 4 | 382 | 0.94 | 2.81 | 2.54 | 0 | 0 | 2 | 5 | 7 |
| MPA | 5 | 1444 | 0.78 | 2.87 | 2.55 | 0 | 0 | 2 | 5 | 7 |
| MPA | 6 | 378 | 0.94 | 2.83 | 2.56 | 0 | 0 | 2 | 5 | 7 |
| MPA | 7 | 1809 | 0.75 | 2.72 | 2.49 | 0 | 0 | 2 | 5 | 7 |
| MPA | 8 | 486 | 0.92 | 2.98 | 2.53 | 0 | 0 | 3 | 5 | 7 |
| MPA | 9 | 1397 | 0.79 | 3.05 | 2.54 | 0 | 0 | 3 | 5 | 7 |
| MPA | 10 | 517 | 0.92 | 3.14 | 2.52 | 0 | 0 | 3 | 5 | 7 |
| MPA | 11 | 358 | 0.94 | 3.13 | 2.57 | 0 | 0 | 3 | 5 | 7 |
| VPA | 1 | 1016 | 0.87 | 1.64 | 1.96 | 0 | 0 | 1 | 3 | 7 |
| VPA | 2 | 476 | 0.93 | 1.35 | 1.86 | 0 | 0 | 0 | 2 | 7 |
| VPA | 3 | 844 | 0.88 | 1.24 | 1.79 | 0 | 0 | 0 | 2 | 7 |
| VPA | 4 | 381 | 0.94 | 1.18 | 1.79 | 0 | 0 | 0 | 2 | 7 |
| VPA | 5 | 1443 | 0.78 | 1.19 | 1.78 | 0 | 0 | 0 | 2 | 7 |
| VPA | 6 | 377 | 0.94 | 1.12 | 1.73 | 0 | 0 | 0 | 2 | 7 |
| VPA | 7 | 1807 | 0.75 | 1.09 | 1.69 | 0 | 0 | 0 | 2 | 7 |
| VPA | 8 | 483 | 0.93 | 1.35 | 1.90 | 0 | 0 | 0 | 2 | 7 |
| VPA | 9 | 1393 | 0.79 | 1.14 | 1.74 | 0 | 0 | 0 | 2 | 7 |
| VPA | 10 | 515 | 0.92 | 1.23 | 1.79 | 0 | 0 | 0 | 2 | 7 |
| VPA | 11 | 353 | 0.94 | 1.20 | 1.76 | 0 | 0 | 0 | 2 | 7 |
| LH | 1 | 994 | 0.87 | 0.73 | 0.44 | 0 | 0 | 1 | 1 | 1 |
| LH | 2 | 459 | 0.93 | 0.73 | 0.44 | 0 | 0 | 1 | 1 | 1 |
| LH | 3 | 821 | 0.88 | 0.71 | 0.46 | 0 | 0 | 1 | 1 | 1 |
| LH | 4 | 350 | 0.94 | 0.69 | 0.46 | 0 | 0 | 1 | 1 | 1 |
| LH | 5 | 1430 | 0.78 | 0.69 | 0.46 | 0 | 0 | 1 | 1 | 1 |
| LH | 6 | 359 | 0.94 | 0.68 | 0.47 | 0 | 0 | 1 | 1 | 1 |
| LH | 7 | 1795 | 0.75 | 0.68 | 0.46 | 0 | 0 | 1 | 1 | 1 |
| LH | 8 | 469 | 0.93 | 0.68 | 0.47 | 0 | 0 | 1 | 1 | 1 |
| LH | 9 | 1368 | 0.80 | 0.68 | 0.47 | 0 | 0 | 1 | 1 | 1 |
| LH | 10 | 494 | 0.92 | 0.68 | 0.47 | 0 | 0 | 1 | 1 | 1 |
| LH | 11 | 329 | 0.94 | 0.67 | 0.47 | 0 | 0 | 1 | 1 | 1 |
| SH | 1 | 960 | 0.87 | 3.15 | 0.76 | 1 | 3 | 3 | 4 | 5 |
| SH | 2 | 456 | 0.93 | 3.18 | 0.76 | 1 | 3 | 3 | 4 | 5 |
| SH | 3 | 822 | 0.88 | 3.13 | 0.76 | 1 | 3 | 3 | 3 | 5 |
| SH | 4 | 347 | 0.94 | 3.10 | 0.76 | 1 | 3 | 3 | 3 | 5 |
| SH | 5 | 1428 | 0.78 | 3.09 | 0.74 | 1 | 3 | 3 | 3 | 5 |
| SH | 6 | 355 | 0.94 | 3.11 | 0.77 | 1 | 3 | 3 | 3 | 5 |
| SH | 7 | 1791 | 0.75 | 3.09 | 0.77 | 1 | 3 | 3 | 3 | 5 |
| SH | 8 | 464 | 0.93 | 3.10 | 0.78 | 1 | 3 | 3 | 3 | 5 |
| SH | 9 | 1366 | 0.80 | 3.09 | 0.78 | 1 | 3 | 3 | 3 | 5 |
| SH | 10 | 491 | 0.92 | 3.14 | 0.81 | 1 | 3 | 3 | 4 | 5 |
| SH | 11 | 324 | 0.94 | 3.12 | 0.79 | 1 | 3 | 3 | 4 | 5 |
| LS | 1 | 919 | 0.88 | 7.57 | 1.40 | 0 | 7 | 8 | 8 | 10 |
| LS | 2 | 844 | 0.87 | 7.51 | 1.38 | 0 | 7 | 8 | 8 | 10 |
| LS | 3 | 952 | 0.86 | 7.47 | 1.37 | 0 | 7 | 8 | 8 | 10 |
| LS | 4 | 828 | 0.86 | 7.45 | 1.34 | 0 | 7 | 8 | 8 | 10 |
| LS | 5 | 633 | 0.90 | 7.45 | 1.36 | 0 | 7 | 8 | 8 | 10 |
| LS | 6 | 1035 | 0.83 | 7.47 | 1.38 | 0 | 7 | 8 | 8 | 10 |
| LS | 7 | 687 | 0.90 | 7.32 | 1.43 | 0 | 7 | 8 | 8 | 10 |
| LS | 8 | 555 | 0.91 | 7.42 | 1.38 | 0 | 7 | 8 | 8 | 10 |
| LS | 9 | 768 | 0.89 | 7.39 | 1.41 | 0 | 7 | 8 | 8 | 10 |
| LS | 10 | 738 | 0.89 | 7.39 | 1.39 | 0 | 7 | 8 | 8 | 10 |
| LS | 11 | 750 | 0.87 | 7.33 | 1.49 | 0 | 7 | 8 | 8 | 10 |
| *Note.* LPA = light physical activity; MPA = moderate physical activity; VPA = vigorous physical activity; LH = long-standing health issues SH = self-rated health; LS = life satisfaction. | | | | | | | | | | |

| **Table 6**  *Descriptives and Distributions for Light Physical Activity, Moderate Physical Activity, Vigorous Physical Activity, Long-Standing Health Issues, Subjective Health, and Life Satisfaction at All Time Points – UKHLS Data* | | | | | | | | | | |
| --- | --- | --- | --- | --- | --- | --- | --- | --- | --- | --- |
| Variable | Wave | n_Missing | Complete Rate | *M* | *SD* | 0th Percentile | 25th Percentile | 50th Percentile | 75th Percentile | 100th Percentile |
| LPA | 1 | 3054 | 0.93 | 4.33 | 2.70 | 0 | 2 | 5 | 7 | 7 |
| LPA | 2 | 1357 | 0.96 | 4.62 | 2.59 | 0 | 2 | 5 | 7 | 7 |
| LPA | 3 | 750 | 0.98 | 4.58 | 2.58 | 0 | 2 | 5 | 7 | 7 |
| LPA | 4 | 524 | 0.98 | 4.62 | 2.53 | 0 | 3 | 5 | 7 | 7 |
| MPA | 1 | 3163 | 0.92 | 1.71 | 2.35 | 0 | 0 | 0 | 3 | 7 |
| MPA | 2 | 1478 | 0.96 | 1.91 | 2.41 | 0 | 0 | 1 | 3 | 7 |
| MPA | 3 | 939 | 0.97 | 2.03 | 2.45 | 0 | 0 | 1 | 4 | 7 |
| MPA | 4 | 712 | 0.97 | 1.98 | 2.42 | 0 | 0 | 1 | 4 | 7 |
| VPA | 1 | 3041 | 0.93 | 1.25 | 1.98 | 0 | 0 | 0 | 2 | 7 |
| VPA | 2 | 1326 | 0.96 | 1.44 | 2.03 | 0 | 0 | 0 | 3 | 7 |
| VPA | 3 | 748 | 0.98 | 1.55 | 2.10 | 0 | 0 | 0 | 3 | 7 |
| VPA | 4 | 539 | 0.98 | 1.45 | 2.03 | 0 | 0 | 0 | 3 | 7 |
| LH | 1 | 63 | 1.00 | 0.67 | 0.47 | 0 | 0 | 1 | 1 | 1 |
| LH | 2 | 40 | 1.00 | 0.64 | 0.48 | 0 | 0 | 1 | 1 | 1 |
| LH | 3 | 95 | 1.00 | 0.64 | 0.48 | 0 | 0 | 1 | 1 | 1 |
| LH | 4 | 99 | 1.00 | 0.68 | 0.47 | 0 | 0 | 1 | 1 | 1 |
| SH | 1 | 4487 | 0.89 | 3.39 | 1.07 | 1 | 3 | 3 | 4 | 5 |
| SH | 2 | 2470 | 0.93 | 3.29 | 1.04 | 1 | 3 | 3 | 4 | 5 |
| SH | 3 | 1232 | 0.96 | 3.24 | 1.03 | 1 | 3 | 3 | 4 | 5 |
| SH | 4 | 396 | 0.99 | 3.24 | 1.03 | 1 | 3 | 3 | 4 | 5 |
| LS | 1 | 4535 | 0.89 | 5.26 | 1.45 | 1 | 5 | 6 | 6 | 7 |
| LS | 2 | 2606 | 0.93 | 5.15 | 1.49 | 1 | 4 | 6 | 6 | 7 |
| LS | 3 | 1384 | 0.96 | 5.14 | 1.40 | 1 | 4 | 6 | 6 | 7 |
| LS | 4 | 560 | 0.98 | 5.16 | 1.40 | 1 | 4 | 6 | 6 | 7 |
| *Note.* LPA = light physical activity; MPA = moderate physical activity; VPA = vigorous physical activity; HC = long-standing health issues; SH = self-rated health; LS = life satisfaction. | | | | | | | | | | |

| **Table 7**  *Parameters and Model Fit Indices for the Random Intercept Cross-Lagged Panel Model – HILDA Data* | | | | |
| --- | --- | --- | --- | --- |
|  | RI-CLPM | | |  |
|  | Est. | *SE* | *p* | Stand. Est […] |
| $\gamma_{PA\sim PA}$ | 0.269 | 0.003 | <.001 | [0.269, 0.298] |
| $\beta_{PA\sim LH}$ | 0.077 | 0.009 | <.001 | [0.021, 0.022] |
| $\beta_{PA\sim SH}$ | 0.089 | 0.005 | <.001 | [0.045, 0.053] |
| $\beta_{PA\sim LS}$ | 0.020 | 0.003 | <.001 | [0.017, 0.022] |
| $\gamma_{LH\sim LH}$ | 0.242 | 0.004 | <.001 | [0.242, 0.254] |
| $\beta_{LH\sim PA}$ | 0.005 | 0.001 | <.001 | [0.018, 0.020] |
| $\beta_{LH\sim SH}$ | 0.041 | 0.001 | <.001 | [0.077, 0.092] |
| $\beta_{LH\sim LS}$ | 0.006 | 0.001 | <.001 | [0.019, 0.024] |
| $\gamma_{SH\sim SH}$ | 0.254 | 0.003 | <.001 | [0.254, 0.298] |
| $\beta_{SH\sim LH}$ | 0.128 | 0.005 | <.001 | [0.068, 0.070] |
| $\beta_{SH\sim PA}$ | 0.030 | 0.001 | <.001 | [0.060, 0.066] |
| $\beta_{SH\sim LS}$ | 0.032 | 0.001 | <.001 | [0.056, 0.070] |
| $\gamma_{LS\sim LS}$ | 0.252 | 0.004 | <.001 | [0.252, 0.316] |
| $\beta_{LS\sim LH}$ | 0.063 | 0.008 | <.001 | [0.019, 0.020] |
| $\beta_{LS\sim PA}$ | 0.023 | 0.002 | <.001 | [0.026, 0.029] |
| $\beta_{LS\sim SH}$ | 0.091 | 0.005 | <.001 | [0.052, 0.061] |
| $T_{PA\sim\sim LH}$ | 0.107 | 0.003 | <.001 | 0.332 |
| $T_{PA\sim\sim SH}$ | 0.368 | 0.006 | <.001 | 0.468 |
| $T_{PA\sim\sim LS}$ | 0.205 | 0.009 | <.001 | 0.197 |
| $T_{LH\sim\sim SH}$ | 0.172 | 0.002 | <.001 | 0.695 |
| $T_{LH\sim\sim LS}$ | 0.079 | 0.003 | <.001 | 0.241 |
| $T_{SH\sim\sim LS}$ | 0.353 | 0.007 | <.001 | 0.441 |
|  |  |  |  |  |
| AIC | 2798555.181  2800437.145  .955  .955  .027 [.027, .028]  .046  3870  744 | |  |  |
| BIC |  |  |  |  |
| Robust CFI |  |  |  |  |
| Robust TLI |  |  |  |  |
| Robust RMSEA [90% CI] |  |  |  |  |
| SRMR |  |  |  |  |
| Degrees of freedom |  |  |  |  |
| Number of parameters |  |  |  |  |
| *Note.* $\gamma$ = autoregressive effects; $\beta$ = cross-lagged effects;$T$ = random intercept factors; PA = moderate-to-vigorous physical activity; LH = long-standing health issues; SH = self-rated health; LS = life satisfaction; [ ] = describes the values between which the standardized effects vary. | | | | |

**Table 8**

*Correlational Table of Moderate-to-Vigorous Physical Activity between the Different Measurement Points*

| Variable | 1 | 2 | 3 | 4 | 5 | 6 | 7 | 8 | 9 | 10 | 11 | 12 | 13 | 14 | 15 | 16 | 17 | 18 | 19 | 20 | 21 |
| --- | --- | --- | --- | --- | --- | --- | --- | --- | --- | --- | --- | --- | --- | --- | --- | --- | --- | --- | --- | --- | --- |
|  |  |  |  |  |  |  |  |  |  |  |  |  |  |  |  |  |  |  |  |  |  |
| 1. PA_1 |  |  |  |  |  |  |  |  |  |  |  |  |  |  |  |  |  |  |  |  |  |
|  |  |  |  |  |  |  |  |  |  |  |  |  |  |  |  |  |  |  |  |  |  |
| 2. PA_2 | .53** |  |  |  |  |  |  |  |  |  |  |  |  |  |  |  |  |  |  |  |  |
|  |  |  |  |  |  |  |  |  |  |  |  |  |  |  |  |  |  |  |  |  |  |
| 3. PA_3 | .48** | .55** |  |  |  |  |  |  |  |  |  |  |  |  |  |  |  |  |  |  |  |
|  |  |  |  |  |  |  |  |  |  |  |  |  |  |  |  |  |  |  |  |  |  |
| 4. PA_4 | .47** | .51** | .57** |  |  |  |  |  |  |  |  |  |  |  |  |  |  |  |  |  |  |
|  |  |  |  |  |  |  |  |  |  |  |  |  |  |  |  |  |  |  |  |  |  |
| 5. PA_5 | .42** | .47** | .52** | .57** |  |  |  |  |  |  |  |  |  |  |  |  |  |  |  |  |  |
|  |  |  |  |  |  |  |  |  |  |  |  |  |  |  |  |  |  |  |  |  |  |
| 6. PA_6 | .40** | .45** | .48** | .50** | .56** |  |  |  |  |  |  |  |  |  |  |  |  |  |  |  |  |
|  |  |  |  |  |  |  |  |  |  |  |  |  |  |  |  |  |  |  |  |  |  |
| 7. PA_7 | .40** | .43** | .45** | .48** | .52** | .56** |  |  |  |  |  |  |  |  |  |  |  |  |  |  |  |
|  |  |  |  |  |  |  |  |  |  |  |  |  |  |  |  |  |  |  |  |  |  |
| 8. PA_8 | .37** | .42** | .44** | .45** | .49** | .53** | .57** |  |  |  |  |  |  |  |  |  |  |  |  |  |  |
|  |  |  |  |  |  |  |  |  |  |  |  |  |  |  |  |  |  |  |  |  |  |
| 9. PA_9 | .36** | .40** | .42** | .43** | .46** | .49** | .53** | .58** |  |  |  |  |  |  |  |  |  |  |  |  |  |
|  |  |  |  |  |  |  |  |  |  |  |  |  |  |  |  |  |  |  |  |  |  |
| 10. PA_10 | .36** | .40** | .42** | .44** | .46** | .48** | .51** | .54** | .59** |  |  |  |  |  |  |  |  |  |  |  |  |
|  |  |  |  |  |  |  |  |  |  |  |  |  |  |  |  |  |  |  |  |  |  |
| 11. PA_11 | .35** | .38** | .41** | .40** | .44** | .45** | .48** | .51** | .54** | .59** |  |  |  |  |  |  |  |  |  |  |  |
|  |  |  |  |  |  |  |  |  |  |  |  |  |  |  |  |  |  |  |  |  |  |
| 12. PA_12 | .35** | .36** | .39** | .39** | .42** | .43** | .45** | .50** | .51** | .55** | .59** |  |  |  |  |  |  |  |  |  |  |
|  |  |  |  |  |  |  |  |  |  |  |  |  |  |  |  |  |  |  |  |  |  |
| 13. PA_13 | .32** | .35** | .36** | .36** | .39** | .41** | .40** | .44** | .46** | .50** | .52** | .56** |  |  |  |  |  |  |  |  |  |
|  |  |  |  |  |  |  |  |  |  |  |  |  |  |  |  |  |  |  |  |  |  |
| 14. PA_14 | .34** | .35** | .38** | .39** | .40** | .41** | .43** | .46** | .46** | .49** | .51** | .54** | .57** |  |  |  |  |  |  |  |  |
|  |  |  |  |  |  |  |  |  |  |  |  |  |  |  |  |  |  |  |  |  |  |
| 15. PA_15 | .32** | .33** | .36** | .37** | .38** | .39** | .41** | .44** | .46** | .47** | .48** | .51** | .52** | .60** |  |  |  |  |  |  |  |
|  |  |  |  |  |  |  |  |  |  |  |  |  |  |  |  |  |  |  |  |  |  |
| 16. PA_16 | .32** | .35** | .35** | .36** | .38** | .39** | .39** | .42** | .43** | .46** | .47** | .49** | .49** | .55** | .60** |  |  |  |  |  |  |
|  |  |  |  |  |  |  |  |  |  |  |  |  |  |  |  |  |  |  |  |  |  |
| 17. PA_17 | .29** | .31** | .32** | .33** | .35** | .35** | .38** | .40** | .41** | .43** | .44** | .46** | .46** | .51** | .55** | .58** |  |  |  |  |  |
|  |  |  |  |  |  |  |  |  |  |  |  |  |  |  |  |  |  |  |  |  |  |
| 18. PA_18 | .28** | .31** | .33** | .33** | .37** | .37** | .38** | .41** | .42** | .43** | .44** | .45** | .45** | .48** | .52** | .55** | .58** |  |  |  |  |
|  |  |  |  |  |  |  |  |  |  |  |  |  |  |  |  |  |  |  |  |  |  |
| 19. PA_19 | .28** | .30** | .32** | .34** | .36** | .36** | .36** | .39** | .41** | .42** | .42** | .44** | .44** | .47** | .50** | .51** | .55** | .61** |  |  |  |
|  |  |  |  |  |  |  |  |  |  |  |  |  |  |  |  |  |  |  |  |  |  |
| 20. PA_20 | .27** | .30** | .30** | .33** | .34** | .35** | .37** | .38** | .39** | .41** | .41** | .40** | .41** | .43** | .46** | .47** | .49** | .53** | .57** |  |  |
|  |  |  |  |  |  |  |  |  |  |  |  |  |  |  |  |  |  |  |  |  |  |
| 21. PA_21 | .25** | .27** | .27** | .29** | .30** | .32** | .33** | .33** | .36** | .38** | .38** | .39** | .39** | .42** | .43** | .45** | .46** | .50** | .53** | .59** |  |
|  |  |  |  |  |  |  |  |  |  |  |  |  |  |  |  |  |  |  |  |  |  |
| 22. PA_22 | .27** | .28** | .29** | .30** | .32** | .34** | .35** | .37** | .39** | .39** | .39** | .40** | .39** | .42** | .43** | .46** | .47** | .49** | .53** | .54** | .58** |
|  |  |  |  |  |  |  |  |  |  |  |  |  |  |  |  |  |  |  |  |  |  |

*Note.* PA = moderate-to-vigorous physical activity; * indicates *p* < .05. ** indicates *p* < .01.

**Table 9**

*Correlational Table of Long-Standing Health Issues between the Different Measurement Points*

| Variable | 1 | 2 | 3 | 4 | 5 | 6 | 7 | 8 | 9 | 10 | 11 | 12 | 13 | 14 | 15 | 16 | 17 | 18 | 19 | 20 | 21 |
| --- | --- | --- | --- | --- | --- | --- | --- | --- | --- | --- | --- | --- | --- | --- | --- | --- | --- | --- | --- | --- | --- |
|  |  |  |  |  |  |  |  |  |  |  |  |  |  |  |  |  |  |  |  |  |  |
| 1. LH_1 |  |  |  |  |  |  |  |  |  |  |  |  |  |  |  |  |  |  |  |  |  |
|  |  |  |  |  |  |  |  |  |  |  |  |  |  |  |  |  |  |  |  |  |  |
| 2. LH_2 | .57** |  |  |  |  |  |  |  |  |  |  |  |  |  |  |  |  |  |  |  |  |
|  |  |  |  |  |  |  |  |  |  |  |  |  |  |  |  |  |  |  |  |  |  |
| 3. LH_3 | .53** | .58** |  |  |  |  |  |  |  |  |  |  |  |  |  |  |  |  |  |  |  |
|  |  |  |  |  |  |  |  |  |  |  |  |  |  |  |  |  |  |  |  |  |  |
| 4. LH_4 | .51** | .56** | .61** |  |  |  |  |  |  |  |  |  |  |  |  |  |  |  |  |  |  |
|  |  |  |  |  |  |  |  |  |  |  |  |  |  |  |  |  |  |  |  |  |  |
| 5. LH_5 | .49** | .52** | .57** | .60** |  |  |  |  |  |  |  |  |  |  |  |  |  |  |  |  |  |
|  |  |  |  |  |  |  |  |  |  |  |  |  |  |  |  |  |  |  |  |  |  |
| 6. LH_6 | .47** | .51** | .52** | .57** | .60** |  |  |  |  |  |  |  |  |  |  |  |  |  |  |  |  |
|  |  |  |  |  |  |  |  |  |  |  |  |  |  |  |  |  |  |  |  |  |  |
| 7. LH_7 | .45** | .49** | .52** | .54** | .57** | .62** |  |  |  |  |  |  |  |  |  |  |  |  |  |  |  |
|  |  |  |  |  |  |  |  |  |  |  |  |  |  |  |  |  |  |  |  |  |  |
| 8. LH_8 | .43** | .46** | .50** | .53** | .53** | .58** | .62** |  |  |  |  |  |  |  |  |  |  |  |  |  |  |
|  |  |  |  |  |  |  |  |  |  |  |  |  |  |  |  |  |  |  |  |  |  |
| 9. LH_9 | .42** | .45** | .48** | .50** | .52** | .55** | .58** | .60** |  |  |  |  |  |  |  |  |  |  |  |  |  |
|  |  |  |  |  |  |  |  |  |  |  |  |  |  |  |  |  |  |  |  |  |  |
| 10. LH_10 | .40** | .44** | .46** | .49** | .50** | .52** | .56** | .57** | .62** |  |  |  |  |  |  |  |  |  |  |  |  |
|  |  |  |  |  |  |  |  |  |  |  |  |  |  |  |  |  |  |  |  |  |  |
| 11. LH_11 | .40** | .43** | .45** | .47** | .50** | .52** | .55** | .58** | .59** | .64** |  |  |  |  |  |  |  |  |  |  |  |
|  |  |  |  |  |  |  |  |  |  |  |  |  |  |  |  |  |  |  |  |  |  |
| 12. LH_12 | .39** | .42** | .43** | .45** | .48** | .50** | .53** | .55** | .56** | .60** | .62** |  |  |  |  |  |  |  |  |  |  |
|  |  |  |  |  |  |  |  |  |  |  |  |  |  |  |  |  |  |  |  |  |  |
| 13. LH_13 | .38** | .41** | .41** | .45** | .46** | .48** | .51** | .53** | .55** | .55** | .58** | .62** |  |  |  |  |  |  |  |  |  |
|  |  |  |  |  |  |  |  |  |  |  |  |  |  |  |  |  |  |  |  |  |  |
| 14. LH_14 | .39** | .40** | .43** | .45** | .47** | .48** | .51** | .52** | .54** | .57** | .57** | .60** | .63** |  |  |  |  |  |  |  |  |
|  |  |  |  |  |  |  |  |  |  |  |  |  |  |  |  |  |  |  |  |  |  |
| 15. LH_15 | .36** | .37** | .40** | .42** | .43** | .45** | .47** | .50** | .52** | .53** | .56** | .58** | .59** | .65** |  |  |  |  |  |  |  |
|  |  |  |  |  |  |  |  |  |  |  |  |  |  |  |  |  |  |  |  |  |  |
| 16. LH_16 | .36** | .38** | .39** | .42** | .44** | .45** | .46** | .48** | .51** | .51** | .54** | .57** | .57** | .62** | .66** |  |  |  |  |  |  |
|  |  |  |  |  |  |  |  |  |  |  |  |  |  |  |  |  |  |  |  |  |  |
| 17. LH_17 | .35** | .36** | .37** | .40** | .41** | .41** | .43** | .46** | .47** | .47** | .49** | .52** | .53** | .58** | .60** | .63** |  |  |  |  |  |
|  |  |  |  |  |  |  |  |  |  |  |  |  |  |  |  |  |  |  |  |  |  |
| 18. LH_18 | .35** | .37** | .39** | .40** | .41** | .42** | .44** | .45** | .48** | .48** | .51** | .53** | .53** | .57** | .60** | .63** | .64** |  |  |  |  |
|  |  |  |  |  |  |  |  |  |  |  |  |  |  |  |  |  |  |  |  |  |  |
| 19. LH_19 | .33** | .34** | .37** | .39** | .40** | .40** | .42** | .45** | .46** | .46** | .49** | .50** | .50** | .54** | .55** | .57** | .59** | .65** |  |  |  |
|  |  |  |  |  |  |  |  |  |  |  |  |  |  |  |  |  |  |  |  |  |  |
| 20. LH_20 | .31** | .34** | .37** | .38** | .40** | .39** | .42** | .43** | .45** | .45** | .48** | .50** | .50** | .53** | .55** | .57** | .58** | .61** | .63** |  |  |
|  |  |  |  |  |  |  |  |  |  |  |  |  |  |  |  |  |  |  |  |  |  |
| 21. LH_21 | .31** | .32** | .35** | .37** | .38** | .36** | .40** | .40** | .43** | .44** | .44** | .44** | .46** | .48** | .50** | .52** | .53** | .56** | .59** | .62** |  |
|  |  |  |  |  |  |  |  |  |  |  |  |  |  |  |  |  |  |  |  |  |  |
| 22. LH_22 | .31** | .33** | .36** | .35** | .37** | .37** | .41** | .41** | .42** | .43** | .44** | .45** | .45** | .49** | .50** | .51** | .52** | .55** | .57** | .59** | .60** |
|  |  |  |  |  |  |  |  |  |  |  |  |  |  |  |  |  |  |  |  |  |  |

*Note.* LH = long-standing health issues; * indicates *p* < .05. ** indicates *p* < .01.

**Table 10**

*Correlational Table of Self-Rated Health between the Different Measurement Points*

| Variable | 1 | 2 | 3 | 4 | 5 | 6 | 7 | 8 | 9 | 10 | 11 | 12 | 13 | 14 | 15 | 16 | 17 | 18 | 19 | 20 | 21 |
| --- | --- | --- | --- | --- | --- | --- | --- | --- | --- | --- | --- | --- | --- | --- | --- | --- | --- | --- | --- | --- | --- |
|  |  |  |  |  |  |  |  |  |  |  |  |  |  |  |  |  |  |  |  |  |  |
| 1. SH_1 |  |  |  |  |  |  |  |  |  |  |  |  |  |  |  |  |  |  |  |  |  |
|  |  |  |  |  |  |  |  |  |  |  |  |  |  |  |  |  |  |  |  |  |  |
| 2. SH_2 | .69** |  |  |  |  |  |  |  |  |  |  |  |  |  |  |  |  |  |  |  |  |
|  |  |  |  |  |  |  |  |  |  |  |  |  |  |  |  |  |  |  |  |  |  |
| 3. SH_3 | .65** | .71** |  |  |  |  |  |  |  |  |  |  |  |  |  |  |  |  |  |  |  |
|  |  |  |  |  |  |  |  |  |  |  |  |  |  |  |  |  |  |  |  |  |  |
| 4. SH_4 | .63** | .67** | .72** |  |  |  |  |  |  |  |  |  |  |  |  |  |  |  |  |  |  |
|  |  |  |  |  |  |  |  |  |  |  |  |  |  |  |  |  |  |  |  |  |  |
| 5. SH_5 | .60** | .67** | .69** | .72** |  |  |  |  |  |  |  |  |  |  |  |  |  |  |  |  |  |
|  |  |  |  |  |  |  |  |  |  |  |  |  |  |  |  |  |  |  |  |  |  |
| 6. SH_6 | .60** | .64** | .67** | .69** | .72** |  |  |  |  |  |  |  |  |  |  |  |  |  |  |  |  |
|  |  |  |  |  |  |  |  |  |  |  |  |  |  |  |  |  |  |  |  |  |  |
| 7. SH_7 | .58** | .63** | .64** | .67** | .69** | .73** |  |  |  |  |  |  |  |  |  |  |  |  |  |  |  |
|  |  |  |  |  |  |  |  |  |  |  |  |  |  |  |  |  |  |  |  |  |  |
| 8. SH_8 | .56** | .62** | .63** | .64** | .66** | .70** | .73** |  |  |  |  |  |  |  |  |  |  |  |  |  |  |
|  |  |  |  |  |  |  |  |  |  |  |  |  |  |  |  |  |  |  |  |  |  |
| 9. SH_9 | .55** | .60** | .61** | .62** | .64** | .67** | .69** | .72** |  |  |  |  |  |  |  |  |  |  |  |  |  |
|  |  |  |  |  |  |  |  |  |  |  |  |  |  |  |  |  |  |  |  |  |  |
| 10. SH_10 | .53** | .59** | .59** | .60** | .63** | .65** | .67** | .69** | .72** |  |  |  |  |  |  |  |  |  |  |  |  |
|  |  |  |  |  |  |  |  |  |  |  |  |  |  |  |  |  |  |  |  |  |  |
| 11. SH_11 | .53** | .58** | .60** | .60** | .63** | .64** | .65** | .67** | .70** | .73** |  |  |  |  |  |  |  |  |  |  |  |
|  |  |  |  |  |  |  |  |  |  |  |  |  |  |  |  |  |  |  |  |  |  |
| 12. SH_12 | .51** | .57** | .57** | .58** | .61** | .63** | .64** | .65** | .67** | .70** | .72** |  |  |  |  |  |  |  |  |  |  |
|  |  |  |  |  |  |  |  |  |  |  |  |  |  |  |  |  |  |  |  |  |  |
| 13. SH_13 | .51** | .55** | .57** | .57** | .59** | .61** | .62** | .63** | .65** | .67** | .68** | .71** |  |  |  |  |  |  |  |  |  |
|  |  |  |  |  |  |  |  |  |  |  |  |  |  |  |  |  |  |  |  |  |  |
| 14. SH_14 | .51** | .56** | .57** | .58** | .59** | .61** | .62** | .63** | .64** | .65** | .67** | .69** | .72** |  |  |  |  |  |  |  |  |
|  |  |  |  |  |  |  |  |  |  |  |  |  |  |  |  |  |  |  |  |  |  |
| 15. SH_15 | .50** | .55** | .56** | .57** | .59** | .60** | .61** | .62** | .63** | .65** | .66** | .67** | .69** | .72** |  |  |  |  |  |  |  |
|  |  |  |  |  |  |  |  |  |  |  |  |  |  |  |  |  |  |  |  |  |  |
| 16. SH_16 | .48** | .53** | .54** | .55** | .56** | .58** | .58** | .61** | .62** | .63** | .64** | .65** | .66** | .70** | .72** |  |  |  |  |  |  |
|  |  |  |  |  |  |  |  |  |  |  |  |  |  |  |  |  |  |  |  |  |  |
| 17. SH_17 | .48** | .52** | .54** | .54** | .55** | .57** | .58** | .58** | .60** | .61** | .62** | .63** | .65** | .68** | .68** | .71** |  |  |  |  |  |
|  |  |  |  |  |  |  |  |  |  |  |  |  |  |  |  |  |  |  |  |  |  |
| 18. SH_18 | .48** | .53** | .53** | .53** | .56** | .57** | .58** | .58** | .60** | .61** | .61** | .62** | .64** | .66** | .68** | .70** | .73** |  |  |  |  |
|  |  |  |  |  |  |  |  |  |  |  |  |  |  |  |  |  |  |  |  |  |  |
| 19. SH_19 | .47** | .50** | .52** | .52** | .54** | .55** | .57** | .57** | .58** | .60** | .60** | .61** | .62** | .65** | .65** | .67** | .69** | .73** |  |  |  |
|  |  |  |  |  |  |  |  |  |  |  |  |  |  |  |  |  |  |  |  |  |  |
| 20. SH_20 | .48** | .51** | .52** | .53** | .55** | .56** | .56** | .57** | .59** | .59** | .59** | .60** | .62** | .63** | .64** | .66** | .67** | .70** | .72** |  |  |
|  |  |  |  |  |  |  |  |  |  |  |  |  |  |  |  |  |  |  |  |  |  |
| 21. SH_21 | .45** | .50** | .50** | .52** | .52** | .53** | .55** | .55** | .57** | .57** | .57** | .58** | .59** | .62** | .63** | .64** | .66** | .68** | .69** | .73** |  |
|  |  |  |  |  |  |  |  |  |  |  |  |  |  |  |  |  |  |  |  |  |  |
| 22. SH_22 | .44** | .49** | .50** | .50** | .51** | .52** | .54** | .54** | .57** | .57** | .57** | .57** | .58** | .61** | .62** | .62** | .64** | .66** | .67** | .69** | .73** |
|  |  |  |  |  |  |  |  |  |  |  |  |  |  |  |  |  |  |  |  |  |  |

*Note.* SH = self-rated health; * indicates *p* < .05. ** indicates *p* < .01.

**Table 11**

*Correlational Table of Life Satisfaction between the Different Measurement Points*

| Variable | 1 | 2 | 3 | 4 | 5 | 6 | 7 | 8 | 9 | 10 | 11 | 12 | 13 | 14 | 15 | 16 | 17 | 18 | 19 | 20 | 21 |
| --- | --- | --- | --- | --- | --- | --- | --- | --- | --- | --- | --- | --- | --- | --- | --- | --- | --- | --- | --- | --- | --- |
|  |  |  |  |  |  |  |  |  |  |  |  |  |  |  |  |  |  |  |  |  |  |
| 1. LS_1 |  |  |  |  |  |  |  |  |  |  |  |  |  |  |  |  |  |  |  |  |  |
|  |  |  |  |  |  |  |  |  |  |  |  |  |  |  |  |  |  |  |  |  |  |
| 2. LS_2 | .55** |  |  |  |  |  |  |  |  |  |  |  |  |  |  |  |  |  |  |  |  |
|  |  |  |  |  |  |  |  |  |  |  |  |  |  |  |  |  |  |  |  |  |  |
| 3. LS_3 | .52** | .56** |  |  |  |  |  |  |  |  |  |  |  |  |  |  |  |  |  |  |  |
|  |  |  |  |  |  |  |  |  |  |  |  |  |  |  |  |  |  |  |  |  |  |
| 4. LS_4 | .49** | .53** | .60** |  |  |  |  |  |  |  |  |  |  |  |  |  |  |  |  |  |  |
|  |  |  |  |  |  |  |  |  |  |  |  |  |  |  |  |  |  |  |  |  |  |
| 5. LS_5 | .47** | .49** | .55** | .59** |  |  |  |  |  |  |  |  |  |  |  |  |  |  |  |  |  |
|  |  |  |  |  |  |  |  |  |  |  |  |  |  |  |  |  |  |  |  |  |  |
| 6. LS_6 | .45** | .47** | .50** | .54** | .59** |  |  |  |  |  |  |  |  |  |  |  |  |  |  |  |  |
|  |  |  |  |  |  |  |  |  |  |  |  |  |  |  |  |  |  |  |  |  |  |
| 7. LS_7 | .40** | .45** | .46** | .51** | .54** | .58** |  |  |  |  |  |  |  |  |  |  |  |  |  |  |  |
|  |  |  |  |  |  |  |  |  |  |  |  |  |  |  |  |  |  |  |  |  |  |
| 8. LS_8 | .42** | .44** | .46** | .49** | .54** | .55** | .59** |  |  |  |  |  |  |  |  |  |  |  |  |  |  |
|  |  |  |  |  |  |  |  |  |  |  |  |  |  |  |  |  |  |  |  |  |  |
| 9. LS_9 | .42** | .45** | .46** | .48** | .52** | .51** | .53** | .58** |  |  |  |  |  |  |  |  |  |  |  |  |  |
|  |  |  |  |  |  |  |  |  |  |  |  |  |  |  |  |  |  |  |  |  |  |
| 10. LS_10 | .39** | .42** | .44** | .46** | .48** | .49** | .50** | .55** | .60** |  |  |  |  |  |  |  |  |  |  |  |  |
|  |  |  |  |  |  |  |  |  |  |  |  |  |  |  |  |  |  |  |  |  |  |
| 11. LS_11 | .39** | .42** | .44** | .45** | .49** | .50** | .50** | .54** | .57** | .61** |  |  |  |  |  |  |  |  |  |  |  |
|  |  |  |  |  |  |  |  |  |  |  |  |  |  |  |  |  |  |  |  |  |  |
| 12. LS_12 | .38** | .40** | .42** | .43** | .47** | .47** | .49** | .51** | .54** | .58** | .61** |  |  |  |  |  |  |  |  |  |  |
|  |  |  |  |  |  |  |  |  |  |  |  |  |  |  |  |  |  |  |  |  |  |
| 13. LS_13 | .38** | .40** | .41** | .44** | .46** | .46** | .48** | .50** | .51** | .56** | .56** | .61** |  |  |  |  |  |  |  |  |  |
|  |  |  |  |  |  |  |  |  |  |  |  |  |  |  |  |  |  |  |  |  |  |
| 14. LS_14 | .37** | .40** | .43** | .44** | .45** | .45** | .46** | .49** | .51** | .53** | .53** | .59** | .62** |  |  |  |  |  |  |  |  |
|  |  |  |  |  |  |  |  |  |  |  |  |  |  |  |  |  |  |  |  |  |  |
| 15. LS_15 | .37** | .38** | .41** | .43** | .45** | .44** | .45** | .47** | .48** | .51** | .51** | .55** | .57** | .63** |  |  |  |  |  |  |  |
|  |  |  |  |  |  |  |  |  |  |  |  |  |  |  |  |  |  |  |  |  |  |
| 16. LS_16 | .35** | .36** | .39** | .41** | .42** | .43** | .43** | .46** | .48** | .49** | .49** | .52** | .55** | .58** | .63** |  |  |  |  |  |  |
|  |  |  |  |  |  |  |  |  |  |  |  |  |  |  |  |  |  |  |  |  |  |
| 17. LS_17 | .35** | .35** | .39** | .40** | .42** | .41** | .43** | .44** | .46** | .48** | .47** | .50** | .52** | .55** | .58** | .63** |  |  |  |  |  |
|  |  |  |  |  |  |  |  |  |  |  |  |  |  |  |  |  |  |  |  |  |  |
| 18. LS_18 | .35** | .37** | .41** | .40** | .42** | .43** | .42** | .44** | .45** | .45** | .48** | .51** | .51** | .55** | .56** | .58** | .62** |  |  |  |  |
|  |  |  |  |  |  |  |  |  |  |  |  |  |  |  |  |  |  |  |  |  |  |
| 19. LS_19 | .32** | .36** | .38** | .38** | .40** | .40** | .41** | .44** | .44** | .45** | .45** | .49** | .48** | .52** | .54** | .55** | .59** | .63** |  |  |  |
|  |  |  |  |  |  |  |  |  |  |  |  |  |  |  |  |  |  |  |  |  |  |
| 20. LS_20 | .33** | .35** | .37** | .37** | .38** | .39** | .39** | .41** | .43** | .44** | .44** | .47** | .47** | .50** | .50** | .52** | .54** | .58** | .61** |  |  |
|  |  |  |  |  |  |  |  |  |  |  |  |  |  |  |  |  |  |  |  |  |  |
| 21. LS_21 | .34** | .34** | .35** | .37** | .40** | .37** | .37** | .39** | .42** | .41** | .41** | .43** | .45** | .47** | .49** | .49** | .51** | .53** | .55** | .61** |  |
|  |  |  |  |  |  |  |  |  |  |  |  |  |  |  |  |  |  |  |  |  |  |
| 22. LS_22 | .33** | .33** | .34** | .37** | .37** | .36** | .39** | .40** | .41** | .41** | .42** | .45** | .44** | .48** | .49** | .49** | .52** | .53** | .55** | .57** | .61** |
|  |  |  |  |  |  |  |  |  |  |  |  |  |  |  |  |  |  |  |  |  |  |

*Note.* LS = life satisfaction; * indicates *p* < .05. ** indicates *p* < .01.

| **Table 12**  *Parameters and Model Fit Indices for the Random Intercept Cross-Lagged Panel Model – SOEP Data* | | | | |
| --- | --- | --- | --- | --- |
|  | RI-CLPM | | |  |
|  | Est. | *SE* | *p* | Stand. Est […] |
| $\gamma_{PA\sim PA}$ | 0.250 | 0.004 | <.001 | [0.195, 0.282] |
| $\beta_{PA\sim LH}$ | –0.018 | 0.014 | .198 | [–0.003, –0.006] |
| $\beta_{PA\sim SH}$ | 0.025 | 0.004 | <.001 | [0.014, 0.020] |
| $\beta_{PA\sim LS}$ | 0.006 | 0.002 | .001 | [0.007, 0.011] |
| $\gamma_{LH\sim LH}$ | 0.036 | 0.005 | <.001 | [0.033, 0.045] |
| $\beta_{LH\sim PA}$ | 0.000 | 0.001 | .997 | 0.000 |
| $\beta_{LH\sim SH}$ | 0.013 | 0.001 | <.001 | [0.039, 0.044] |
| $\beta_{LH\sim LS}$ | 0.003 | 0.001 | <.001 | [0.015, 0.019] |
| $\gamma_{SH\sim SH}$ | 0.189 | 0.004 | <.001 | [0.179, 0.201] |
| $\beta_{SH\sim LH}$ | 0.093 | 0.011 | <.001 | [0.028, 0.041] |
| $\beta_{SH\sim PA}$ | 0.016 | 0.002 | <.001 | [0.020, 0.027] |
| $\beta_{SH\sim LS}$ | 0.025 | 0.002 | <.001 | [0.045, 0.056] |
| $\gamma_{LS\sim LS}$ | 0.186 | 0.004 | <.001 | [0.179, 0.195] |
| $\beta_{LS\sim LH}$ | 0.072 | 0.023 | .001 | [0.011, 0.015] |
| $\beta_{LS\sim PA}$ | 0.016 | 0.004 | <.001 | [0.010, 0.014] |
| $\beta_{LS\sim SH}$ | 0.115 | 0.006 | <.001 | [0.054, 0.061] |
| $T_{PA\sim\sim LH}$ | 0.025 | 0.001 | <.001 | 0.369 |
| $T_{PA\sim\sim SH}$ | 0.303 | 0.004 | <.001 | 0.438 |
| $T_{PA\sim\sim LS}$ | 0.333 | 0.007 | <.001 | 0.304 |
| $T_{LH\sim\sim SH}$ | 0.035 | 0.001 | <.001 | 0.673 |
| $T_{LH\sim\sim LS}$ | 0.032 | 0.001 | <.001 | 0.394 |
| $T_{SH\sim\sim LS}$ | 0.493 | 0.006 | <.001 | 0.583 |
|  |  |  |  |  |
| AIC | 2895149.803  2897764.083  .963  .960  .026 [.025, .027]  .039  1430  472 | |  |  |
| BIC |  |  |  |  |
| Robust CFI |  |  |  |  |
| Robust TLI |  |  |  |  |
| Robust RMSEA [90% CI] |  |  |  |  |
| SRMR |  |  |  |  |
| Degrees of freedom |  |  |  |  |
| Number of parameters |  |  |  |  |
| *Note.* $\gamma$ = autoregressive effects; $\beta$ = cross-lagged effects;$T$ = random intercept factors; PA = sports participation; LH = long-standing health issues; SH = self-rated health; LS = life satisfaction; [ ] = describes the values between which the standardized effects vary. | | | | |

**Table 13**

*Correlational Table of Sport Participation between the Different Measurement Points*

| Variable | 1 | 2 | 3 | 4 | 5 | 6 | 7 | 8 | 9 | 10 | 11 | 12 | 13 |
| --- | --- | --- | --- | --- | --- | --- | --- | --- | --- | --- | --- | --- | --- |
|  |  |  |  |  |  |  |  |  |  |  |  |  |  |
| 1. PA_1 |  |  |  |  |  |  |  |  |  |  |  |  |  |
|  |  |  |  |  |  |  |  |  |  |  |  |  |  |
| 2. PA_2 | .62** |  |  |  |  |  |  |  |  |  |  |  |  |
|  |  |  |  |  |  |  |  |  |  |  |  |  |  |
| 3. PA_3 | .58** | .66** |  |  |  |  |  |  |  |  |  |  |  |
|  |  |  |  |  |  |  |  |  |  |  |  |  |  |
| 4. PA_4 | .54** | .59** | .66** |  |  |  |  |  |  |  |  |  |  |
|  |  |  |  |  |  |  |  |  |  |  |  |  |  |
| 5. PA_5 | .50** | .53** | .57** | .61** |  |  |  |  |  |  |  |  |  |
|  |  |  |  |  |  |  |  |  |  |  |  |  |  |
| 6. PA_6 | .49** | .52** | .56** | .58** | .64** |  |  |  |  |  |  |  |  |
|  |  |  |  |  |  |  |  |  |  |  |  |  |  |
| 7. PA_7 | .46** | .50** | .52** | .54** | .59** | .67** |  |  |  |  |  |  |  |
|  |  |  |  |  |  |  |  |  |  |  |  |  |  |
| 8. PA_8 | .43** | .46** | .48** | .50** | .54** | .60** | .66** |  |  |  |  |  |  |
|  |  |  |  |  |  |  |  |  |  |  |  |  |  |
| 9. PA_9 | .42** | .44** | .45** | .47** | .52** | .57** | .62** | .67** |  |  |  |  |  |
|  |  |  |  |  |  |  |  |  |  |  |  |  |  |
| 10. PA_10 | .38** | .38** | .41** | .42** | .47** | .48** | .53** | .55** | .57** |  |  |  |  |
|  |  |  |  |  |  |  |  |  |  |  |  |  |  |
| 11. PA_11 | .39** | .39** | .42** | .43** | .46** | .48** | .52** | .55** | .56** | .57** |  |  |  |
|  |  |  |  |  |  |  |  |  |  |  |  |  |  |
| 12. PA_12 | .36** | .38** | .39** | .41** | .45** | .47** | .49** | .52** | .52** | .52** | .60** |  |  |
|  |  |  |  |  |  |  |  |  |  |  |  |  |  |
| 13. PA_13 | .36** | .34** | .36** | .36** | .41** | .42** | .44** | .47** | .46** | .48** | .50** | .53** |  |
|  |  |  |  |  |  |  |  |  |  |  |  |  |  |
| 14. PA_14 | .34** | .31** | .30** | .33** | .35** | .36** | .38** | .41** | .40** | .41** | .44** | .45** | .52** |
|  |  |  |  |  |  |  |  |  |  |  |  |  |  |

*Note.* PA = sport participation; * indicates *p* < .05. ** indicates *p* < .01.

**Table 14**

*Correlational Table of Long-Standing Health Issues between the Different Measurement Points*

| Variable | 1 | 2 | 3 | 4 | 5 | 6 | 7 | 8 | 9 | 10 | 11 | 12 | 13 |
| --- | --- | --- | --- | --- | --- | --- | --- | --- | --- | --- | --- | --- | --- |
|  |  |  |  |  |  |  |  |  |  |  |  |  |  |
| 1. LH_1 |  |  |  |  |  |  |  |  |  |  |  |  |  |
|  |  |  |  |  |  |  |  |  |  |  |  |  |  |
| 2. LH_2 | .15** |  |  |  |  |  |  |  |  |  |  |  |  |
|  |  |  |  |  |  |  |  |  |  |  |  |  |  |
| 3. LH_3 | .12** | .07** |  |  |  |  |  |  |  |  |  |  |  |
|  |  |  |  |  |  |  |  |  |  |  |  |  |  |
| 4. LH_4 | .05** | .10** | .14** |  |  |  |  |  |  |  |  |  |  |
|  |  |  |  |  |  |  |  |  |  |  |  |  |  |
| 5. LH_5 | .04 | .07** | .10** | .10** |  |  |  |  |  |  |  |  |  |
|  |  |  |  |  |  |  |  |  |  |  |  |  |  |
| 6. LH_6 | .03 | .05** | .07** | .08** | .08** |  |  |  |  |  |  |  |  |
|  |  |  |  |  |  |  |  |  |  |  |  |  |  |
| 7. LH_7 | .02 | .03 | .04** | .08** | .09** | .06** |  |  |  |  |  |  |  |
|  |  |  |  |  |  |  |  |  |  |  |  |  |  |
| 8. LH_8 | .04 | .08** | .06** | .07** | .06** | .08** | .10** |  |  |  |  |  |  |
|  |  |  |  |  |  |  |  |  |  |  |  |  |  |
| 9. LH_9 | .02 | .01 | .10** | .04** | .07** | .07** | .08** | .10** |  |  |  |  |  |
|  |  |  |  |  |  |  |  |  |  |  |  |  |  |
| 10. LH_10 | .06 | .10** | .06** | .04** | .07** | .05** | .06** | .13** | .13** |  |  |  |  |
|  |  |  |  |  |  |  |  |  |  |  |  |  |  |
| 11. LH_11 | .03 | .08** | .05* | .04* | .08** | .07** | .06** | .09** | .08** | .12** |  |  |  |
|  |  |  |  |  |  |  |  |  |  |  |  |  |  |
| 12. LH_12 | .01 | –.02 | .03 | .08** | .11** | .05** | .06** | .08** | .09** | .09** | .11** |  |  |
|  |  |  |  |  |  |  |  |  |  |  |  |  |  |
| 13. LH_13 | –.01 | –.02 | –.01 | .03 | .09** | .05** | .07** | .13** | .06** | .08** | .10** | .13** |  |
|  |  |  |  |  |  |  |  |  |  |  |  |  |  |
| 14. LH_14 | .01 | .03 | .04 | .01 | .06** | .03 | .01 | .07** | .08** | .06** | .07** | .10** | .14** |
|  |  |  |  |  |  |  |  |  |  |  |  |  |  |

*Note.* LH = long-standing health issues; * indicates *p* < .05. ** indicates *p* < .01.

**Table 15**

*Correlational Table of Self-Rated Health between the Different Measurement Points*

| Variable | 1 | 2 | 3 | 4 | 5 | 6 | 7 | 8 | 9 | 10 | 11 | 12 | 13 |
| --- | --- | --- | --- | --- | --- | --- | --- | --- | --- | --- | --- | --- | --- |
|  |  |  |  |  |  |  |  |  |  |  |  |  |  |
| 1. SH_1 |  |  |  |  |  |  |  |  |  |  |  |  |  |
|  |  |  |  |  |  |  |  |  |  |  |  |  |  |
| 2. SH_2 | .61** |  |  |  |  |  |  |  |  |  |  |  |  |
|  |  |  |  |  |  |  |  |  |  |  |  |  |  |
| 3. SH_3 | .54** | .61** |  |  |  |  |  |  |  |  |  |  |  |
|  |  |  |  |  |  |  |  |  |  |  |  |  |  |
| 4. SH_4 | .51** | .56** | .61** |  |  |  |  |  |  |  |  |  |  |
|  |  |  |  |  |  |  |  |  |  |  |  |  |  |
| 5. SH_5 | .50** | .54** | .56** | .62** |  |  |  |  |  |  |  |  |  |
|  |  |  |  |  |  |  |  |  |  |  |  |  |  |
| 6. SH_6 | .48** | .52** | .53** | .57** | .62** |  |  |  |  |  |  |  |  |
|  |  |  |  |  |  |  |  |  |  |  |  |  |  |
| 7. SH_7 | .45** | .49** | .51** | .53** | .58** | .63** |  |  |  |  |  |  |  |
|  |  |  |  |  |  |  |  |  |  |  |  |  |  |
| 8. SH_8 | .43** | .46** | .46** | .49** | .54** | .58** | .62** |  |  |  |  |  |  |
|  |  |  |  |  |  |  |  |  |  |  |  |  |  |
| 9. SH_9 | .39** | .44** | .44** | .48** | .51** | .54** | .58** | .63** |  |  |  |  |  |
|  |  |  |  |  |  |  |  |  |  |  |  |  |  |
| 10. SH_10 | .39** | .42** | .43** | .45** | .48** | .51** | .55** | .58** | .59** |  |  |  |  |
|  |  |  |  |  |  |  |  |  |  |  |  |  |  |
| 11. SH_11 | .39** | .40** | .42** | .44** | .45** | .49** | .52** | .55** | .54** | .60** |  |  |  |
|  |  |  |  |  |  |  |  |  |  |  |  |  |  |
| 12. SH_12 | .39** | .42** | .40** | .42** | .43** | .46** | .49** | .52** | .51** | .55** | .60** |  |  |
|  |  |  |  |  |  |  |  |  |  |  |  |  |  |
| 13. SH_13 | .40** | .41** | .39** | .41** | .43** | .45** | .47** | .51** | .48** | .52** | .55** | .59** |  |
|  |  |  |  |  |  |  |  |  |  |  |  |  |  |
| 14. SH_14 | .40** | .41** | .39** | .40** | .41** | .43** | .44** | .48** | .46** | .49** | .50** | .53** | .58** |
|  |  |  |  |  |  |  |  |  |  |  |  |  |  |

*Note.* SH = self-rated health; * indicates *p* < .05. ** indicates *p* < .01.

**Table 16**

*Correlational Table of Life Satisfaction between the Different Measurement Points*

| Variable | 1 | 2 | 3 | 4 | 5 | 6 | 7 | 8 | 9 | 10 | 11 | 12 | 13 |
| --- | --- | --- | --- | --- | --- | --- | --- | --- | --- | --- | --- | --- | --- |
|  |  |  |  |  |  |  |  |  |  |  |  |  |  |
| 1. LS_1 |  |  |  |  |  |  |  |  |  |  |  |  |  |
|  |  |  |  |  |  |  |  |  |  |  |  |  |  |
| 2. LS_2 | .52** |  |  |  |  |  |  |  |  |  |  |  |  |
|  |  |  |  |  |  |  |  |  |  |  |  |  |  |
| 3. LS_3 | .46** | .53** |  |  |  |  |  |  |  |  |  |  |  |
|  |  |  |  |  |  |  |  |  |  |  |  |  |  |
| 4. LS_4 | .44** | .48** | .53** |  |  |  |  |  |  |  |  |  |  |
|  |  |  |  |  |  |  |  |  |  |  |  |  |  |
| 5. LS_5 | .41** | .42** | .48** | .53** |  |  |  |  |  |  |  |  |  |
|  |  |  |  |  |  |  |  |  |  |  |  |  |  |
| 6. LS_6 | .40** | .42** | .44** | .48** | .54** |  |  |  |  |  |  |  |  |
|  |  |  |  |  |  |  |  |  |  |  |  |  |  |
| 7. LS_7 | .38** | .41** | .42** | .45** | .51** | .56** |  |  |  |  |  |  |  |
|  |  |  |  |  |  |  |  |  |  |  |  |  |  |
| 8. LS_8 | .37** | .39** | .39** | .40** | .46** | .50** | .56** |  |  |  |  |  |  |
|  |  |  |  |  |  |  |  |  |  |  |  |  |  |
| 9. LS_9 | .37** | .37** | .38** | .39** | .44** | .47** | .51** | .57** |  |  |  |  |  |
|  |  |  |  |  |  |  |  |  |  |  |  |  |  |
| 10. LS_10 | .35** | .36** | .36** | .38** | .41** | .45** | .47** | .51** | .56** |  |  |  |  |
|  |  |  |  |  |  |  |  |  |  |  |  |  |  |
| 11. LS_11 | .34** | .32** | .37** | .38** | .40** | .43** | .45** | .49** | .50** | .53** |  |  |  |
|  |  |  |  |  |  |  |  |  |  |  |  |  |  |
| 12. LS_12 | .33** | .33** | .35** | .36** | .38** | .43** | .43** | .48** | .47** | .48** | .54** |  |  |
|  |  |  |  |  |  |  |  |  |  |  |  |  |  |
| 13. LS_13 | .35** | .34** | .35** | .35** | .37** | .44** | .42** | .45** | .45** | .45** | .50** | .50** |  |
|  |  |  |  |  |  |  |  |  |  |  |  |  |  |
| 14. LS_14 | .29** | .28** | .29** | .29** | .32** | .34** | .34** | .37** | .38** | .40** | .42** | .41** | .46** |
|  |  |  |  |  |  |  |  |  |  |  |  |  |  |

*Note.* LS = life satisfaction; * indicates *p* < .05. ** indicates *p* < .01.

| **Table 17**  *Parameters and Model Fit Indices for the Random Intercept Cross-Lagged Panel Model – LISS Data* | | | | |
| --- | --- | --- | --- | --- |
|  | RI-CLPM | | |  |
|  | Est. | *SE* | *p* | Stand. Est […] |
| $\gamma_{LPA\sim LPA}$ | 0.135 | 0.007 | <.001 | [0.134, 0.135] |
| $\beta_{LPA\sim MPA}$ | 0.009 | 0.006 | .134 | [0.008, 0.009] |
| $\beta_{LPA\sim VPA}$ | –0.006 | 0.009 | .467 | [–0.004, –0.005] |
| $\beta_{LPA\sim LH}$ | 0.047 | 0.054 | .388 | 0.007 |
| $\beta_{LPA\sim SH}$ | –0.003 | 0.026 | .917 | –0.001 |
| $\beta_{LPA\sim LS}$ | 0.024 | 0.013 | .072 | [0.011, 0.012] |
| $\gamma_{MPA\sim MPA}$ | 0.095 | 0.007 | <.001 | [0.095, 0.096] |
| $\beta_{MPA\sim LPA}$ | 0.016 | 0.006 | .003 | [0.016, 0.017] |
| $\beta_{MPA\sim VPA}$ | 0.044 | 0.009 | <.001 | [0.030, 0.034] |
| $\beta_{MPA\sim LH}$ | 0.062 | 0.051 | .229 | 0.009 |
| $\beta_{MPA\sim SH}$ | 0.025 | 0.024 | .305 | [0.006, 0.007] |
| $\beta_{MPA\sim LS}$ | 0.020 | 0.013 | .116 | [0.009, 0.010] |
| $\gamma_{VPA\sim VPA}$ | 0.112 | 0.008 | <.001 | [0.112, 0.129] |
| $\beta_{VPA\sim MPA}$ | 0.018 | 0.004 | <.001 | 0.027 |
| $\beta_{VPA\sim LPA}$ | 0.002 | 0.004 | .674 | 0.002 |
| $\beta_{VPA\sim LH}$ | 0.029 | 0.034 | .390 | 0.006 |
| $\beta_{VPA\sim SH}$ | 0.063 | 0.017 | <.001 | [0.023, 0.025] |
| $\beta_{VPA\sim LS}$ | 0.010 | 0.009 | .248 | [0.007, 0.008] |
| $\gamma_{LH\sim LH}$ | 0.701 | 0.012 | <.001 | [0.701, 0.706] |
| $\beta_{LH\sim MPA}$ | 0.001 | 0.001 | .181 | 0.006 |
| $\beta_{LH\sim LPA}$ | 0.000 | 0.001 | .462 | 0.003 |
| $\beta_{LH\sim VPA}$ | –0.002 | 0.001 | .029 | [–0.009, –0.011] |
| $\beta_{LH\sim SH}$ | 0.007 | 0.003 | .009 | [0.012, 0.013] |
| $\beta_{LH\sim LS}$ | 0.006 | 0.002 | <.001 | [0.018, 0.020] |
| $\gamma_{SH\sim SH}$ | 0.203 | 0.009 | <.001 | [0.203, 0.223] |
| $\beta_{SH\sim MPA}$ | 0.005 | 0.001 | <.001 | 0.021 |
| $\beta_{SH\sim LPA}$ | 0.002 | 0.001 | .097 | 0.009 |
| $\beta_{SH\sim VPA}$ | 0.007 | 0.002 | .001 | [0.020, 0.023] |
| $\beta_{SH\sim LH}$ | 0.095 | 0.014 | <.001 | 0.058 |
| $\beta_{SH\sim LS}$ | 0.049 | 0.004 | <.001 | [0.094, 0.102] |
| $\gamma_{LS\sim LS}$ | 0.249 | 0.012 | <.001 | [0.249, 0.270] |
| $\beta_{LS\sim MPA}$ | 0.006 | 0.003 | .040 | 0.012 |
| $\beta_{LS\sim LPA}$ | 0.005 | 0.003 | .105 | 0.010 |
| $\beta_{LS\sim VPA}$ | 0.004 | 0.004 | .329 | [0.006, 0.007] |
| $\beta_{LS\sim LH}$ | 0.056 | 0.026 | .034 | 0.018 |
| $\beta_{LS\sim SH}$ | 0.085 | 0.014 | <.001 | [0.044, 0.049] |
| $T_{LPA\sim\sim MPA}$ | 1.030 | 0.035 | <.001 | 0.427 |
| $T_{LPA\sim\sim VPA}$ | 0.334 | 0.026 | <.001 | 0.181 |
| $T_{LPA\sim\sim LH}$ | 0.023 | 0.008 | .004 | 0.044 |
| $T_{LPA\sim\sim SH}$ | 0.095 | 0.013 | <.001 | 0.100 |
| $T_{LPA\sim\sim LS}$ | 0.189 | 0.023 | <.001 | 0.115 |
| $T_{MPA\sim\sim VPA}$ | 0.860 | 0.027 | <.001 | 0.486 |
| $T_{MPA\sim\sim LH}$ | 0.041 | 0.008 | <.001 | 0.081 |
| $T_{MPA\sim\sim SH}$ | 0.198 | 0.012 | <.001 | 0.216 |
| $T_{MPA\sim\sim LS}$ | 0.102 | 0.022 | <.001 | 0.065 |
| $T_{VPA\sim\sim LH}$ | 0.074 | 0.005 | <.001 | 0.188 |
| $T_{VPA\sim\sim SH}$ | 0.194 | 0.009 | <.001 | 0.278 |
| $T_{VPA\sim\sim LS}$ | 0.060 | 0.016 | <.001 | 0.050 |
| $T_{LH\sim\sim SH}$ | 0.119 | 0.003 | <.001 | 0.592 |
| $T_{LH\sim\sim LS}$ | 0.067 | 0.005 | <.001 | 0.192 |
| $T_{SH\sim\sim LS}$ | 0.287 | 0.009 | <.001 | 0.460 |
|  |  |  |  |  |
| AIC | 1162992.5841164763.592.980  .980  .020 [.019, .021]  .027  2112  746 | |  |  |
| BIC |  | |  |  |
| Robust CFI |  | |  |  |
| Robust TLI |  | |  |  |
| Robust RMSEA [90% CI] |  | |  |  |
| SRMR |  | |  |  |
| Degrees of freedom |  | |  |  |
| Number of parameters |  | |  |  |
| *Note.* $\gamma$ = autoregressive effects; $\beta$ = cross-lagged effects;$T$ = random intercept factors; LPA = light physical activity; MPA = moderate physical activity; VPA = vigorous physical activity; LH = long-standing health issues; SH = self-rated health; LS = life satisfaction; [ ] = describes the values between which the standardized effects vary. | | | | |

**Table 18**

*Correlational Table of Light Physical Activity between the Different Measurement Points*

| Variable | 1 | 2 | 3 | 4 | 5 | 6 | 7 | 8 | 9 | 10 |
| --- | --- | --- | --- | --- | --- | --- | --- | --- | --- | --- |
|  |  |  |  |  |  |  |  |  |  |  |
| 1. LPA_1 |  |  |  |  |  |  |  |  |  |  |
|  |  |  |  |  |  |  |  |  |  |  |
| 2. LPA_2 | .44** |  |  |  |  |  |  |  |  |  |
|  |  |  |  |  |  |  |  |  |  |  |
| 3. LPA_3 | .40** | .45** |  |  |  |  |  |  |  |  |
|  |  |  |  |  |  |  |  |  |  |  |
| 4. LPA_4 | .39** | .41** | .45** |  |  |  |  |  |  |  |
|  |  |  |  |  |  |  |  |  |  |  |
| 5. LPA_5 | .34** | .41** | .42** | .46** |  |  |  |  |  |  |
|  |  |  |  |  |  |  |  |  |  |  |
| 6. LPA_6 | .32** | .40** | .41** | .44** | .45** |  |  |  |  |  |
|  |  |  |  |  |  |  |  |  |  |  |
| 7. LPA_7 | .34** | .36** | .40** | .42** | .45** | .49** |  |  |  |  |
|  |  |  |  |  |  |  |  |  |  |  |
| 8. LPA_8 | .27** | .35** | .35** | .35** | .43** | .42** | .43** |  |  |  |
|  |  |  |  |  |  |  |  |  |  |  |
| 9. LPA_9 | .27** | .33** | .34** | .35** | .38** | .42** | .41** | .44** |  |  |
|  |  |  |  |  |  |  |  |  |  |  |
| 10. LPA_10 | .28** | .33** | .32** | .34** | .37** | .39** | .40** | .42** | .49** |  |
|  |  |  |  |  |  |  |  |  |  |  |
| 11. LPA_11 | .26** | .29** | .32** | .34** | .36** | .37** | .40** | .43** | .46** | .48** |
|  |  |  |  |  |  |  |  |  |  |  |

*Note.* LPA = light physical activity; * indicates *p* < .05. ** indicates *p* < .01.

**Table 19**

*Correlational Table of Moderate Physical Activity between the Different Measurement Points*

| Variable | 1 | 2 | 3 | 4 | 5 | 6 | 7 | 8 | 9 | 10 |
| --- | --- | --- | --- | --- | --- | --- | --- | --- | --- | --- |
|  |  |  |  |  |  |  |  |  |  |  |
| 1. MPA_1 |  |  |  |  |  |  |  |  |  |  |
|  |  |  |  |  |  |  |  |  |  |  |
| 2. MPA_2 | .40** |  |  |  |  |  |  |  |  |  |
|  |  |  |  |  |  |  |  |  |  |  |
| 3. MPA_3 | .39** | .42** |  |  |  |  |  |  |  |  |
|  |  |  |  |  |  |  |  |  |  |  |
| 4. MPA_4 | .37** | .40** | .43** |  |  |  |  |  |  |  |
|  |  |  |  |  |  |  |  |  |  |  |
| 5. MPA_5 | .37** | .38** | .41** | .41** |  |  |  |  |  |  |
|  |  |  |  |  |  |  |  |  |  |  |
| 6. MPA_6 | .32** | .36** | .38** | .40** | .45** |  |  |  |  |  |
|  |  |  |  |  |  |  |  |  |  |  |
| 7. MPA_7 | .32** | .34** | .38** | .37** | .42** | .43** |  |  |  |  |
|  |  |  |  |  |  |  |  |  |  |  |
| 8. MPA_8 | .28** | .31** | .33** | .32** | .38** | .38** | .42** |  |  |  |
|  |  |  |  |  |  |  |  |  |  |  |
| 9. MPA_9 | .29** | .32** | .36** | .34** | .35** | .36** | .38** | .41** |  |  |
|  |  |  |  |  |  |  |  |  |  |  |
| 10. MPA_10 | .32** | .31** | .35** | .32** | .38** | .38** | .38** | .39** | .44** |  |
|  |  |  |  |  |  |  |  |  |  |  |
| 11. MPA_11 | .28** | .26** | .33** | .29** | .36** | .33** | .36** | .36** | .42** | .45** |
|  |  |  |  |  |  |  |  |  |  |  |

*Note.* MPA = moderate physical activity; * indicates *p* < .05. ** indicates *p* < .01.

**Table 20**

*Correlational Table of Vigorous Physical Activity between the Different Measurement Points*

| Variable | 1 | 2 | 3 | 4 | 5 | 6 | 7 | 8 | 9 | 10 |
| --- | --- | --- | --- | --- | --- | --- | --- | --- | --- | --- |
|  |  |  |  |  |  |  |  |  |  |  |
| 1. VPA_1 |  |  |  |  |  |  |  |  |  |  |
|  |  |  |  |  |  |  |  |  |  |  |
| 2. VPA_2 | .46** |  |  |  |  |  |  |  |  |  |
|  |  |  |  |  |  |  |  |  |  |  |
| 3. VPA_3 | .43** | .45** |  |  |  |  |  |  |  |  |
|  |  |  |  |  |  |  |  |  |  |  |
| 4. VPA_4 | .39** | .43** | .45** |  |  |  |  |  |  |  |
|  |  |  |  |  |  |  |  |  |  |  |
| 5. VPA_5 | .39** | .43** | .41** | .49** |  |  |  |  |  |  |
|  |  |  |  |  |  |  |  |  |  |  |
| 6. VPA_6 | .36** | .40** | .40** | .44** | .49** |  |  |  |  |  |
|  |  |  |  |  |  |  |  |  |  |  |
| 7. VPA_7 | .34** | .38** | .40** | .41** | .47** | .47** |  |  |  |  |
|  |  |  |  |  |  |  |  |  |  |  |
| 8. VPA_8 | .34** | .38** | .37** | .40** | .41** | .45** | .46** |  |  |  |
|  |  |  |  |  |  |  |  |  |  |  |
| 9. VPA_9 | .33** | .37** | .35** | .37** | .38** | .40** | .43** | .46** |  |  |
|  |  |  |  |  |  |  |  |  |  |  |
| 10. VPA_10 | .33** | .37** | .39** | .39** | .40** | .40** | .42** | .42** | .49** |  |
|  |  |  |  |  |  |  |  |  |  |  |
| 11. VPA_11 | .29** | .31** | .30** | .34** | .36** | .37** | .40** | .39** | .48** | .46** |
|  |  |  |  |  |  |  |  |  |  |  |

*Note.* VPA = vigorous physical activity; * indicates *p* < .05. ** indicates *p* < .01.

**Table 21**

*Correlational Table of Long-Standing Health Issues between the Different Measurement Points*

| Variable | 1 | 2 | 3 | 4 | 5 | 6 | 7 | 8 | 9 | 10 |
| --- | --- | --- | --- | --- | --- | --- | --- | --- | --- | --- |
|  |  |  |  |  |  |  |  |  |  |  |
| 1. LH_1 |  |  |  |  |  |  |  |  |  |  |
|  |  |  |  |  |  |  |  |  |  |  |
| 2. LH_2 | .85** |  |  |  |  |  |  |  |  |  |
|  |  |  |  |  |  |  |  |  |  |  |
| 3. LH_3 | .78** | .87** |  |  |  |  |  |  |  |  |
|  |  |  |  |  |  |  |  |  |  |  |
| 4. LH_4 | .71** | .79** | .86** |  |  |  |  |  |  |  |
|  |  |  |  |  |  |  |  |  |  |  |
| 5. LH_5 | .67** | .75** | .80** | .88** |  |  |  |  |  |  |
|  |  |  |  |  |  |  |  |  |  |  |
| 6. LH_6 | .64** | .70** | .74** | .79** | .88** |  |  |  |  |  |
|  |  |  |  |  |  |  |  |  |  |  |
| 7. LH_7 | .61** | .67** | .69** | .74** | .81** | .88** |  |  |  |  |
|  |  |  |  |  |  |  |  |  |  |  |
| 8. LH_8 | .58** | .62** | .65** | .70** | .75** | .80** | .88** |  |  |  |
|  |  |  |  |  |  |  |  |  |  |  |
| 9. LH_9 | .56** | .60** | .63** | .67** | .71** | .75** | .81** | .89** |  |  |
|  |  |  |  |  |  |  |  |  |  |  |
| 10. LH_10 | .56** | .61** | .61** | .65** | .69** | .72** | .76** | .81** | .90** |  |
|  |  |  |  |  |  |  |  |  |  |  |
| 11. LH_11 | .54** | .58** | .58** | .62** | .65** | .68** | .72** | .76** | .82** | .89** |
|  |  |  |  |  |  |  |  |  |  |  |

*Note.* LH = long-standing health issues; * indicates *p* < .05. ** indicates *p* < .01.

**Table 22**

*Correlational Table of Self-Rated Health between the Different Measurement Points*

| Variable | 1 | 2 | 3 | 4 | 5 | 6 | 7 | 8 | 9 | 10 |
| --- | --- | --- | --- | --- | --- | --- | --- | --- | --- | --- |
|  |  |  |  |  |  |  |  |  |  |  |
| 1. SH_1 |  |  |  |  |  |  |  |  |  |  |
|  |  |  |  |  |  |  |  |  |  |  |
| 2. SH_2 | .63** |  |  |  |  |  |  |  |  |  |
|  |  |  |  |  |  |  |  |  |  |  |
| 3. SH_3 | .58** | .66** |  |  |  |  |  |  |  |  |
|  |  |  |  |  |  |  |  |  |  |  |
| 4. SH_4 | .57** | .63** | .66** |  |  |  |  |  |  |  |
|  |  |  |  |  |  |  |  |  |  |  |
| 5. SH_5 | .55** | .58** | .62** | .68** |  |  |  |  |  |  |
|  |  |  |  |  |  |  |  |  |  |  |
| 6. SH_6 | .53** | .56** | .60** | .65** | .70** |  |  |  |  |  |
|  |  |  |  |  |  |  |  |  |  |  |
| 7. SH_7 | .51** | .54** | .57** | .62** | .66** | .70** |  |  |  |  |
|  |  |  |  |  |  |  |  |  |  |  |
| 8. SH_8 | .50** | .53** | .57** | .60** | .64** | .67** | .69** |  |  |  |
|  |  |  |  |  |  |  |  |  |  |  |
| 9. SH_9 | .49** | .52** | .55** | .56** | .61** | .63** | .65** | .69** |  |  |
|  |  |  |  |  |  |  |  |  |  |  |
| 10. SH_10 | .48** | .52** | .53** | .56** | .60** | .62** | .64** | .65** | .69** |  |
|  |  |  |  |  |  |  |  |  |  |  |
| 11. SH_11 | .49** | .53** | .52** | .56** | .60** | .59** | .63** | .65** | .66** | .68** |
|  |  |  |  |  |  |  |  |  |  |  |

*Note.* SH = self-rated health; * indicates *p* < .05. ** indicates *p* < .01.

**Table 23**

*Correlational Table of Life Satisfaction between the Different Measurement Points*

| Variable | 1 | 2 | 3 | 4 | 5 | 6 | 7 | 8 | 9 | 10 |
| --- | --- | --- | --- | --- | --- | --- | --- | --- | --- | --- |
|  |  |  |  |  |  |  |  |  |  |  |
| 1. LS_1 |  |  |  |  |  |  |  |  |  |  |
|  |  |  |  |  |  |  |  |  |  |  |
| 2. LS_2 | .60** |  |  |  |  |  |  |  |  |  |
|  |  |  |  |  |  |  |  |  |  |  |
| 3. LS_3 | .58** | .66** |  |  |  |  |  |  |  |  |
|  |  |  |  |  |  |  |  |  |  |  |
| 4. LS_4 | .55** | .62** | .65** |  |  |  |  |  |  |  |
|  |  |  |  |  |  |  |  |  |  |  |
| 5. LS_5 | .50** | .57** | .60** | .65** |  |  |  |  |  |  |
|  |  |  |  |  |  |  |  |  |  |  |
| 6. LS_6 | .48** | .54** | .57** | .62** | .64** |  |  |  |  |  |
|  |  |  |  |  |  |  |  |  |  |  |
| 7. LS_7 | .44** | .50** | .53** | .57** | .59** | .65** |  |  |  |  |
|  |  |  |  |  |  |  |  |  |  |  |
| 8. LS_8 | .46** | .51** | .52** | .55** | .57** | .61** | .68** |  |  |  |
|  |  |  |  |  |  |  |  |  |  |  |
| 9. LS_9 | .41** | .46** | .47** | .51** | .55** | .58** | .61** | .62** |  |  |
|  |  |  |  |  |  |  |  |  |  |  |
| 10. LS_10 | .40** | .46** | .47** | .52** | .52** | .54** | .58** | .61** | .66** |  |
|  |  |  |  |  |  |  |  |  |  |  |
| 11. LS_11 | .42** | .44** | .46** | .50** | .52** | .54** | .55** | .58** | .64** | .66** |
|  |  |  |  |  |  |  |  |  |  |  |

*Note.* LS = life satisfaction; * indicates *p* < .05. ** indicates *p* < .01.

| **Table 24**  *Parameters and Model Fit Indices for the Random Intercept Cross-Lagged Panel Model – UKHLS Data* | | | | |
| --- | --- | --- | --- | --- |
|  | RI-CLPM | | |  |
|  | Est. | *SE* | *p* | Stand. Est […] |
| $\gamma_{LPA\sim LPA}$ | 0.136 | 0.007 | <.001 | [0.136, 0.147] |
| $\beta_{LPA\sim MPA}$ | 0.025 | 0.005 | <.001 | [0.025, 0.026] |
| $\beta_{LPA\sim VPA}$ | 0.029 | 0.007 | <.001 | [0.023, 0.024] |
| $\beta_{LPA\sim LH}$ | 0.015 | 0.033 | .668 | 0.002 |
| $\beta_{LPA\sim SH}$ | 0.073 | 0.020 | <.001 | [0.022, 0.024] |
| $\beta_{LPA\sim LS}$ | 0.041 | 0.010 | <.001 | [0.022, 0.023] |
| $\gamma_{MPA\sim MPA}$ | 0.064 | 0.007 | <.001 | [0.063, 0.064] |
| $\beta_{MPA\sim LPA}$ | 0.030 | 0.005 | <.001 | [0.029, 0.031] |
| $\beta_{MPA\sim VPA}$ | 0.089 | 0.008 | <.001 | [0.067, 0.069] |
| $\beta_{MPA\sim LH}$ | 0.024 | 0.034 | .485 | 0.004 |
| $\beta_{MPA\sim SH}$ | 0.092 | 0.020 | <.001 | [0.027, 0.029] |
| $\beta_{MPA\sim LS}$ | 0.010 | 0.010 | .330 | 0.005 |
| $\gamma_{VPA\sim VPA}$ | 0.120 | 0.008 | <.001 | [0.116, 0.120] |
| $\beta_{VPA\sim MPA}$ | 0.024 | 0.004 | <.001 | 0.031 |
| $\beta_{VPA\sim LPA}$ | 0.010 | 0.004 | .020 | [0.012, 0.013] |
| $\beta_{VPA\sim LH}$ | 0.059 | 0.027 | .030 | 0.012 |
| $\beta_{VPA\sim SH}$ | 0.109 | 0.016 | <.001 | [0.041, 0.044] |
| $\beta_{VPA\sim LS}$ | 0.007 | 0.008 | .360 | 0.005 |
| $\gamma_{LH\sim LH}$ | 0.137 | 0.008 | <.001 | [0.134, 0.137] |
| $\beta_{LH\sim MPA}$ | –0.001 | 0.001 | .085 | –0.009 |
| $\beta_{LH\sim LPA}$ | –0.001 | 0.001 | .188 | –0.007 |
| $\beta_{LH\sim VPA}$ | 0.001 | 0.001 | .509 | [0.003, 0.004] |
| $\beta_{LH\sim SH}$ | 0.035 | 0.003 | <.001 | [0.065, 0.069] |
| $\beta_{LH\sim LS}$ | 0.009 | 0.002 | <.001 | [0.030, 0.031] |
| $\gamma_{SH\sim SH}$ | 0.143 | 0.008 | <.001 | [0.144, 0.152] |
| $\beta_{SH\sim MPA}$ | 0.003 | 0.002 | .042 | 0.011 |
| $\beta_{SH\sim LPA}$ | 0.005 | 0.002 | .003 | [0.017, 0.019] |
| $\beta_{SH\sim VPA}$ | 0.011 | 0.002 | <.001 | [0.029, 0.030] |
| $\beta_{SH\sim LH}$ | 0.063 | 0.011 | <.001 | 0.034 |
| $\beta_{SH\sim LS}$ | 0.026 | 0.003 | <.001 | [0.047, 0.049] |
| $\gamma_{LS\sim LS}$ | 0.105 | 0.008 | <.001 | [0.105, 0.108] |
| $\beta_{LS\sim MPA}$ | 0.000 | 0.003 | .990 | 0.000 |
| $\beta_{LS\sim LPA}$ | 0.006 | 0.003 | .035 | [0.012, 0.013] |
| $\beta_{LS\sim VPA}$ | –0.001 | 0.004 | .894 | –0.001 |
| $\beta_{LS\sim LH}$ | 0.037 | 0.018 | .040 | 0.011 |
| $\beta_{LS\sim SH}$ | 0.072 | 0.011 | <.001 | [0.040, 0.043] |
| $T_{LPA\sim\sim MPA}$ | 0.940 | 0.023 | <.001 | 0.515 |
| $T_{LPA\sim\sim VPA}$ | 0.741 | 0.020 | <.001 | 0.387 |
| $T_{LPA\sim\sim LH}$ | 0.189 | 0.005 | <.001 | 0.354 |
| $T_{LPA\sim\sim SH}$ | 0.543 | 0.012 | <.001 | 0.406 |
| $T_{LPA\sim\sim LS}$ | 0.300 | 0.016 | <.001 | 0.212 |
| $T_{MPA\sim\sim VPA}$ | 1.078 | 0.023 | <.001 | 0.760 |
| $T_{MPA\sim\sim LH}$ | 0.109 | 0.004 | <.001 | 0.276 |
| $T_{MPA\sim\sim SH}$ | 0.344 | 0.0095 | <.001 | 0.347 |
| $T_{MPA\sim\sim LS}$ | 0.174 | 0.012 | <.001 | 0.165 |
| $T_{VPA\sim\sim LH}$ | 0.142 | 0.003 | <.001 | 0.344 |
| $T_{VPA\sim\sim SH}$ | 0.443 | 0.009 | <.001 | 0.427 |
| $T_{VPA\sim\sim LS}$ | 0.153 | 0.011 | <.001 | 0.139 |
| $T_{LH\sim\sim SH}$ | 0.215 | 0.002 | <.001 | 0.744 |
| $T_{LH\sim\sim LS}$ | 0.106 | 0.003 | <.001 | 0.345 |
| $T_{SH\sim\sim LS}$ | 0.441 | 0.007 | <.001 | 0.575 |
|  |  |  |  |  |
| AIC | 2928400.5092929714.751.994  .992  .016 [.015, .017]  .018  201  263 | |  |  |
| BIC |  | |  |  |
| Robust CFI |  | |  |  |
| Robust TLI |  | |  |  |
| Robust RMSEA [90% CI] |  | |  |  |
| SRMR |  | |  |  |
| Degrees of freedom |  | |  |  |
| Number of parameters |  | |  |  |
| *Note.* $\gamma$ = autoregressive effects; $\beta$ = cross-lagged effects;$T$ = random intercept factors; LPA = light physical activity; MPA = moderate physical activity; VPA = vigorous physical activity; LH = long-standing health issues; SH = self-rated health; LS = life satisfaction; [ ] = describes the values between which the standardized effects vary. | | | | |

**Table 25**

*Correlational Table of Light Physical Activity*

*between the Different Measurement Points*

| Variable | 1 | 2 | 3 |
| --- | --- | --- | --- |
|  |  |  |  |
| 1. LPA_1 |  |  |  |
|  |  |  |  |
| 2. LPA_2 | .45** |  |  |
|  |  |  |  |
| 3. LPA_3 | .37** | .47** |  |
|  |  |  |  |
| 4. LPA_4 | .32** | .39** | .45** |
|  |  |  |  |

*Note. LPA = light physical activity;*

** indicates p < .05. ** indicates p < .01.*

**Table 26**

*Correlational Table of Moderate Physical*

*Activity between the Different*

*Measurement Points*

| Variable | 1 | 2 | 3 |
| --- | --- | --- | --- |
|  |  |  |  |
| 1. MPA_1 |  |  |  |
|  |  |  |  |
| 2. MPA_2 | .25** |  |  |
|  |  |  |  |
| 3. MPA_3 | .23** | .33** |  |
|  |  |  |  |
| 4. MPA_4 | .21** | .28** | .33** |
|  |  |  |  |

*Note. MPA = moderate physical activity;*

** indicates p < .05. ** indicates p < .01.*

**Table 27**

*Correlational Table of Vigorous Physical*

*Activity between the Different*

*Measurement Points*

| Variable | 1 | 2 | 3 |
| --- | --- | --- | --- |
|  |  |  |  |
| 1. VPA_1 |  |  |  |
|  |  |  |  |
| 2. VPA_2 | .41** |  |  |
|  |  |  |  |
| 3. VPA_3 | .36** | .46** |  |
|  |  |  |  |
| 4. VPA_4 | .33** | .39** | .44** |
|  |  |  |  |

*Note. VPA = vigorous physical activity;*

** indicates p < .05. ** indicates p < .01.*

**Table 28**

*Correlational Table of Long-Standing Health*

*Issues between the Different*

*Measurement Points*

| Variable | 1 | 2 | 3 |
| --- | --- | --- | --- |
|  |  |  |  |
| 1. LH_1 |  |  |  |
|  |  |  |  |
| 2. LH_2 | .58** |  |  |
|  |  |  |  |
| 3. LH_3 | .52** | .59** |  |
|  |  |  |  |
| 4. LH_4 | .47** | .52** | .57** |
|  |  |  |  |

*Note. LH = long-standing health issues;*

** indicates p < .05. ** indicates p < .01.*

**Table 29**

*Correlational Table of Self-Rated Health*

*between the Different Measurement Points*

| Variable | 1 | 2 | 3 |
| --- | --- | --- | --- |
|  |  |  |  |
| 1. SH_1 |  |  |  |
|  |  |  |  |
| 2. SH_2 | .69** |  |  |
|  |  |  |  |
| 3. SH_3 | .64** | .70** |  |
|  |  |  |  |
| 4. SH_4 | .62** | .66** | .71** |
|  |  |  |  |

*Note. SH = self-rated health;*

** indicates p < .05. ** indicates p < .01.*

**Table 30**

*Correlational Table of Life Satisfaction*

*between the Different Measurement Points*

| Variable | 1 | 2 | 3 |
| --- | --- | --- | --- |
|  |  |  |  |
| 1. LS_1 |  |  |  |
|  |  |  |  |
| 2. LS_2 | .43** |  |  |
|  |  |  |  |
| 3. LS_3 | .40** | .46** |  |
|  |  |  |  |
| 4. LS_4 | .38** | .41** | .50** |
|  |  |  |  |

*Note. LS = life satisfaction;*

** indicates p < .05. ** indicates p < .01.*

**Figure 1**

*Statistical Model*


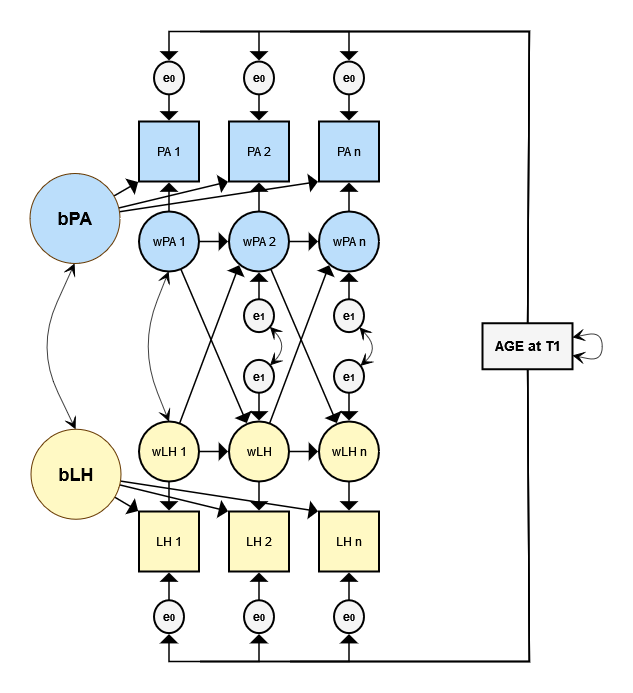


*Note.* Illustration of a random intercept cross-lagged path model (RI-CLPM); Only two predictor variables are shown for simplicity; Preﬁx b denotes between-person associations, and preﬁx w denotes within-person associations; Squares represent observed variables, and circles represent latent variables; Straight arrows represent direct associations, and curved lines represent correlations between variables; PA = physical activity; LH = long-standing health issues.

**References**

Caspersen, C. J., Powell, K. E., & Christenson, G. M. (1985). Physical activity, exercise, and physical ﬁtness: Deﬁnitions and distinctions for health-related research. *Public Health Reports, 100*, 126–131.

Enders, C. K. (2008). A note on the use of missing auxiliary variables in full information maximum likelihood-based structural equation models. *Structural Equation Modeling: A Multidisciplinary Journal, 15*, 434–448. https://doi.org/10.1080/10705510802154307

Enders, C. K. (2010). *Applied missing data analysis.* Guilford Press.

Goebel, J., Grabka, M., Liebig, S., Kroh, M., Richter, D., Schröder, C. & Schupp, J. (2019). The German Socio-Economic Panel (SOEP). *Jahrbücher für Nationalökonomie und Statistik*, *239*, 345–360. https://doi.org/10.1515/jbnst-2018-0022

Graham, J. W. (2003). Adding missing-data-relevant variables to FIML-based structural equation models. *Structural Equation Modeling: A Multidisciplinary Journal, 10*, 80–100. https://doi.org/10.1207/S15328007SEM1001_4

Graham, J. W. (2009). Missing data analysis: Making it work in the real world. *Annual Review of Psychology, 60*, 549–576. https://doi.org/10.1146/annurev.psych.58.110405.085530

Mulder, J. D., & Hamaker, E. L. (2021). Three extensions of the random intercept cross-lagged panel model. *Structural Equation Modeling: A Multidisciplinary Journal, 28*, 638–648. https://doi.org/10.1080/10705511.2020.1784738

Preacher, K. J., & Yaremych, H. E. (2023). Model selection in structural equation modeling. In R. H. Hoyle (Ed.) *Handbook of Structural Equation Modeling* (2nd Ed), pp. 206-222. Guilford.

Scherpenzeel, A.C. & Das, M. (2010). “True” longitudinal and probability-based internet panels: Evidence from the Netherlands. In Das, M., P. Ester, and L. Kaczmirek (Eds.), *Social and Behavioral Research and the Internet: Advances in Applied Methods and Research Strategies.* (pp. 77-104). Boca Raton: Taylor & Francis.

University of Essex, Institute for Social and Economic Research. (2023). *Understanding Society: Waves 1-13, 2009-2022 and Harmonised BHPS: Waves 1-18, 1991-2009. [data collection]. 18th Edition.* UK Data Service. SN: 6614, http://doi.org/10.5255/UKDA-SN-6614-19.

Usami, S., Murayama, K., & Hamaker, E. L. (2019). A unified framework of longitudinal models to examine reciprocal relations. *Psychological Methods, 24*, 637–657. https://doi.org/10.1037/met0000210

Watson, N., & Wooden, M. P. (2012). The HILDA survey: A case study in the design and development of a successful household panel survey. *Longitudinal and Life Course Studies, 3*, 369–381.
